# Integrating "Yang transforming Qi and Yin constituting the body" with immune regulation: an evidence synthesis of multidimensional traditional chinese medicine therapy for immune thrombocytopenia

**DOI:** 10.1186/s13020-025-01318-4

**Published:** 2026-01-09

**Authors:** Xin Zhou, Yang Jiang, Ming Hou, Ningning Shan

**Affiliations:** 1 Department of Hematology, The Second Qilu Hospital of Shandong University, 247 Beiyuan Road, Jinan, 250033 Shandong China; 2 Department of Hematology, Shandong Provincial Hospital Affiliated to Shandong First Medical University, Jinan, 250021 Shandong China; 3 Department of Hematology, Qilu Hospital of Shandong University, Jinan, 250012 Shandong China

**Keywords:** ITP, Traditional Chinese medicine ingredients, Syndrome differentiation and treatment, Traditional Chinese medicine compounds

## Abstract

Immune thrombocytopenia (ITP) is an acquired autoimmune hemorrhagic disorder with a substantial incidence globally across all age groups. Its pathogenesis involves the accelerated immune-mediated platelet destruction and impaired platelet production due to dysfunctional megakaryocyte maturation interactively. ITP is primarily treated by glucocorticoids and intravenous immunoglobulin in Western medicine conventionally. However, these therapies exhibit several limitations such as corticosteroid dependency, increased risk of infection, treatment resistance, and frequent relapse, despite its obvious efficacy in rapidly elevating platelet counts. In contrast, traditional Chinese medicine (TCM) attributes the pathogenesis of ITP (under “blood syndrome” or “purpura disease”) to an imbalance in the fundamental TCM principle of “Yang transforming Qi and Yin constituting the body”. By targeting both pathological platelet destruction and insufficient platelet production, TCM exerts multidimensional therapeutic effects in ITP, with clearly elucidated mechanisms demonstrated by active components from single herbs and compound formulations. The integration of TCM with Western medicine has shown promise in enhancing the therapeutic outcomes of the latter therapy while mitigating their side effects. Accordingly, the present study intends to systematically review the mechanisms of TCM in ITP, summarize recent research advances, analyze current challenges, and propose future research directions. This work is expected to provide potential foundation for further investigation and clinical application of TCM in ITP.

## Introduction

Immune thrombocytopenia (ITP) is an acquired autoimmune hemorrhagic disorder formerly referred to as idiopathic thrombocytopenic purpura, representing one of the more frequently encountered hematological diseases clinically [1, 2]. Currently, glucocorticoids, intravenous immunoglobulin, thrombopoietin receptor agonists, and rituximab are the mainstream for the treatment of ITP in Western medicine [3, 4]. It should be acknowledged that these therapies can effectively increase platelet counts to some extent, but usually associated with adverse effects such as corticosteroid dependence and increased susceptibility to infections in the context of long-term use. Some patients may develop treatment resistance or experience disease relapse.

Traditional Chinese medicine (TCM) has a long-standing history that can date back thousands of years, with the accumulation of extensive clinical experience in hematological disorder treatment. In terms of ITP, TCM has been attached great importance to given its advantages of systemic regulation, minimal side effects, and low recurrence rates [5, 6]. An ITP-related fundamental concept of TCM is the theory of "Yang transforming Qi and Yin constituting the body", which describes the dynamic relationship between functional energy (Qi) and material structure in physiological processes. Yang (positive and active) can promote the transformation of nutrients into functional energy (Qi) that supports metabolism, thermoregulation, and physiological activities. While Yin (substantial and consolidating) can facilitate the materialization of energy into tangible bodily structures (e.g., blood, tissues, and organs). The Yin-Yang balance constitute an important cornerstone for internal homeostasis; and the imbalance of Yin and Yang may induce a series of physical and mental issues. For instance, Yang deficiency may impair energy metabolism and cause hypofunction, while Yin deficiency is often associated with tissue depletion or pathological accumulation. Noticeably, the proposed theoretical framework aligns significantly with modern concepts of immune dysregulation and megakaryocyte dysfunction in ITP [7, 8].

With advancement of modern medical technologies in recent decades, we have acquire adequate evidence for elucidating mechanisms of TCM components in the treatment of ITP, which may offer novel insights and therapeutic strategies for its clinical management. Therefore, this study intends to systematically summarize and review the pathophysiological function of platelets and the pathogenesis of ITP from the perspective of TCM. It is anticipated to offer detailed summary of recent advances concerning the application of various single Chinese herbs, compound Chinese herbal formulations, and integrated traditional Chinese and Western medicine approaches in ITP treatment, so as to lay a foundation for further research and clinical application of TCM in ITP management.

## Pathogenesis of ITP

### Immune-mediated platelet destruction

Currently, thrombocytopenia has been accepted to be a disorder resulted primarily from autoantibody-mediated platelet destruction. GPIIb/IIIa and GPIb-IX-V act as mediating substances for reduced counts of peripheral blood platelets caused by macrophage phagocytosis and platelet destruction [9]. The autoantibody-labeled platelets, whose Fcγ binds to the Fcγ receptor on the surface of macrophages within the spleen, are phagocytosed and destroyed [10]; alternatively, they can induce platelet desialacidification by antibodies, bind to the Ashwell-Morell receptor on the surface of hepatocytes, and be cleared within the liver [11].

**Fig. 1:**
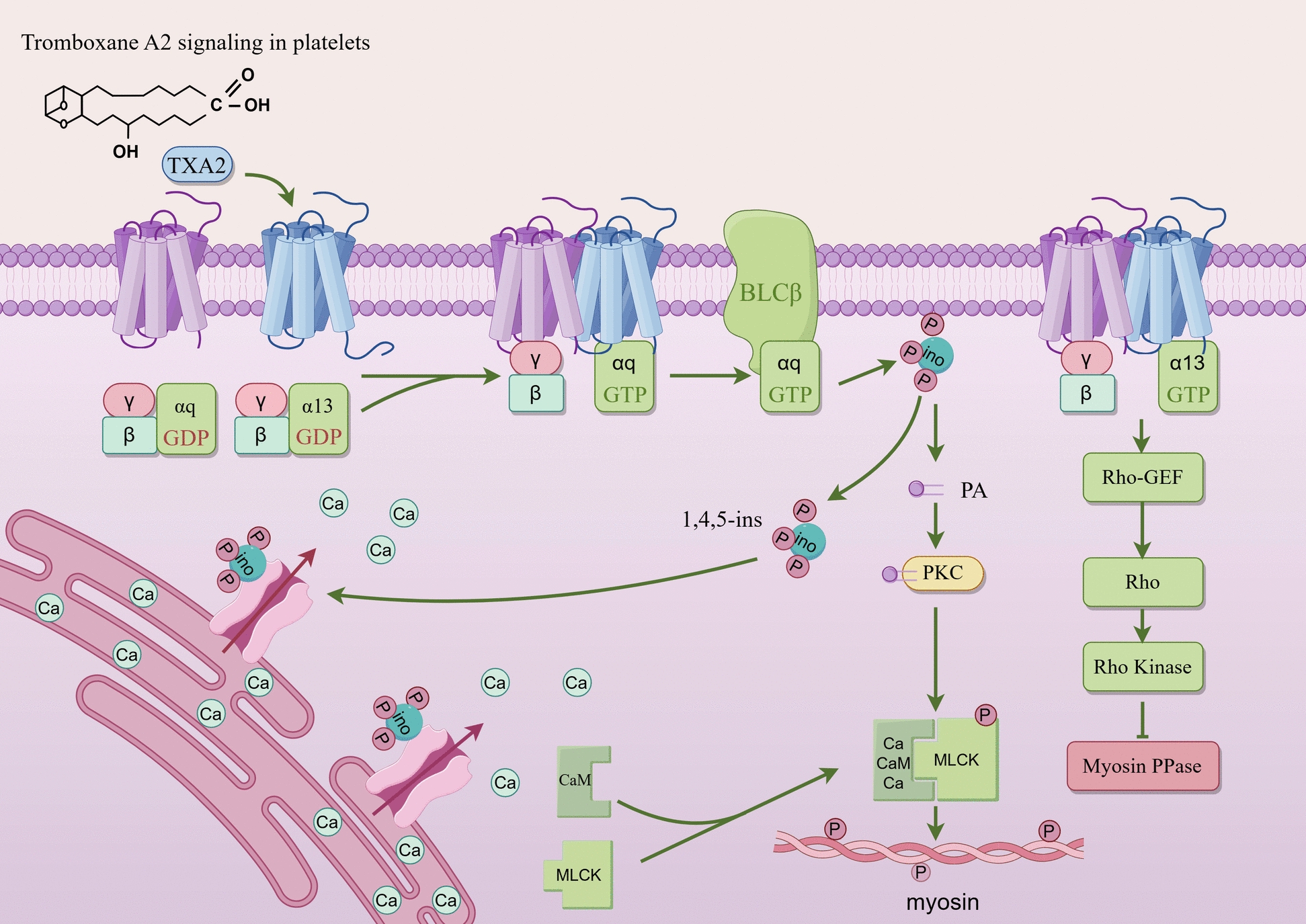
Apoptotic process of platelets and megakaryocytes. Small-molecule inhibitors (e.g., ABT-737, ABT-263, MIK-665, and MC-1) selectively target proteins of the anti-apoptotic Bcl-2 family, including Bcl-xL. Inhibition of Bcl-xL may activate the pro-apoptotic proteins Bak and Bax, resulting in mitochondrial outer membrane permeabilization, caspase-9 activation, and subsequent cleavage of effector caspases-3/7. This apoptotic cascade may induce cell death in megakaryocytes, ultimately impairing platelet production and hemostatic function

Immune dysfunction is a major contributor to the pathogenesis of ITP. Specifically, Bregs can negatively regulate immune dysfunction and inhibit CD19^+^ B cell proliferation, thereby decreasing excessive platelet damage and promoting platelet production [12]. In general, there may be a release of platelet-associated immunoglobulins in the context of B lymphocyte dysfunction. Given their high specificity, these immunoglobulins can activate designated targets on platelet membrane proteins. Moreover, anti-platelet autoantibodies also interfere with the maturation of megakaryocytes, further reducing platelet production and ultimately decreasing the number of platelets in the peripheral blood [13]. Notably, platelet destruction may also involve cytotoxic T cells directly attacking circulating platelets in some patients. This T-cell-mediated cytotoxic effect, together with antibody-mediated mechanisms, constitutes the dual destruction pathway of ITP [14]. For example, platelets may express several functional Toll-like receptors (TLRs) that directly bind to pathogens and mediate anti-infective immunity [15, 16]. Meanwhile, they may also express circulating Fcγ receptor IIA which allows for the interaction with immune complexes [17]. Additionally, platelets express functional CD40 ligand (CD154), which induces endothelial cells to secrete chemokines and express adhesion molecules, thereby facilitating leukocyte recruitment [18]. Platelet factor 4 (PF4/CXCL4) is released to increase neutrophil adhesion to endothelial cells and promote monocyte phagocytosis [19]. Meanwhile, both megakaryocytes and platelets can function as antigen-presenting cells, capable of activating T cells and initiating immune responses [20]. Similar to their parent megakaryocytes, platelets express various immune receptors (e.g., TLRs, immunoglobulin receptors, and costimulatory molecules) and cytokines (e.g., PF4/CXCL4, TGF-β, IL-8, and CXCL1), which are stored in α-granules [21] (Table 1). Activated platelets can release adenosine diphosphate (ADP), thromboxane A2, thrombin, and other autocrine mediators. Figure 1 illustrates the apoptotic pathways in platelets and megakaryocytes, emphasizing the mechanism of programmed cell death influencing thrombopoiesis and hemostatic balance in conditions such as ITP.

**Table 1:** Platelet chemokines/cytokine receptors

Chemokines	Ligands	Functions	References
CCR1	CCL3, CCL5, CCL7, CCL8, and CCL13-16	Significant platelet activation and granule release T cell and monocyte migration Binding to MIP-1-α, RANTES, and less efficiently to MIP-1-β or MCP-1	[22]
CCR3	CCL5, CCL7;CCL11, CCL15-16, CCL24, CCL26	Migration of eosinophils and basophils Signal transduction by increasing intracellular calcium levels	[23], [24]
CCR4	CCL17, and CCL22	High affinity for C–C type chemokines Activity mediated through the activation of the phosphatidylinositol-calcium second messenger system by G (i) proteins	[25]
CXCR1type	CXCL6, CXCL7, and CXCL8	Interleukin-8 receptor Activation of neutrophils	[26]
CXCR4type	CXCL12	MAPK1/MAPK3 activation and signaling cascade Regulation of cell migration (wound healing) LPS-induced inflammatory response, including TNF secretion	[27], [28]
Cytokines			
IFNGR	IFN-γ	Phagocyte activation, antigen presentation, and Th1 cytokine expression Regulation of other cytokines JAK/STAT signaling pathway	[29], [30]
TNFR	TNF-α	TNF-α-Most of TNF-α metabolic effects Regulation of the frequency of effector and/or memory CD4 + or CD8 + T cells by TNF-TNFR	[31]
TGF-βR	TGF-β	Transmembrane ser/thr kinases Cell cycle arrest and wound healing in hematopoietic cells TRAF6 autoubiquitination, and cell death	[32]
IL1R type	IL1-β	PMNS (IL-1,8) and fibroblasts (IL-4) Chemotaxis-adaptor molecule recruitment of TOLLIP, MyD88 and IRAK1 or IRAK2-IL1B Costimulation of IFNG production by mediated Th1 cells	[33]

### Reduced platelet production caused by megakaryocyte maturation disorders

**Fig. 2:**
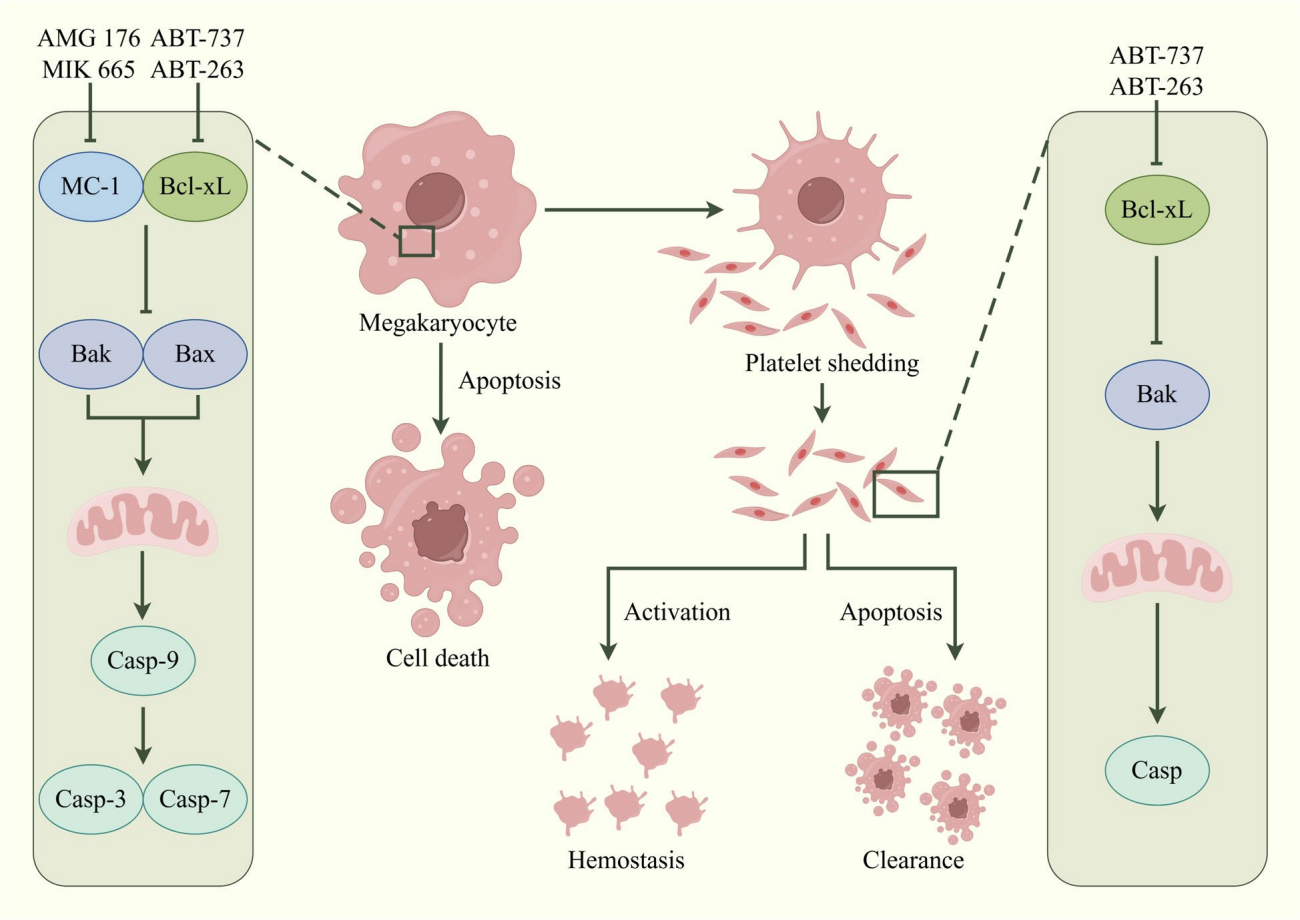
The aggregation process of platelets. Thromboxane A2 binding to its receptor may activate Gαq and Gα13 signaling to induce the activation of phospholipase Cβ. It may increase intracellular calcium levels, activating calcium/calmodulin-dependent kinase and myosin light-chain kinase, thereby promoting myosin light-chain phosphorylation and facilitating platelet aggregation. Diacylglycerol may activate protein kinase C, further enhancing this process. Additionally, Gβγ and Gα13 may activate Rho via Rho guanine nucleotide exchange factors, leading to Rho kinase-mediated inhibition of myosin phosphatase, thereby sustaining platelet aggregation

There may be reduced degree of polyploidization, and defective cytoplasmic maturation, ultimately leading to mitigated platelet production, although there may be normal or even increased number of megakaryocytes in the bone marrow of ITP patients [34]. In addition to attacking circulating platelets, antiplatelet antibodies can also directly inhibit the differentiation and maturation of megakaryocytes by recognizing the same antigenic epitopes on their surface [35]. Hypoxia-inducible factor-1α (HIF-1α) is a key regulator in the development of megakaryocytes. Its expression is downregulated in patients with ITP, resulting in obstructed megakaryocyte maturation. Drugs that stabilize HIF-1α (e.g., Roxadustat) can improve defects in megakaryocyte maturation [36]. Furthermore, an abnormal bone marrow microenvironment is involved in ITP pathogenesis, including abnormal migration and distribution of megakaryocytes as well as imbalanced expression of cell cycle regulatory proteins (e.g., cyclin D1 and P27). These changes may impair platelet production eventually [37]. Recently, some ITP patients have been reported to have abnormal megakaryocyte death, which may be attributed to antibody-dependent cytotoxicity or direct T-cell attack, further exacerbating platelet production insufficiency [35]. Autophagy also acts as a key player in megakaryocyte differentiation, platelet biogenesis, and platelet function. Dysregulated autophagy, such as impaired ATG7 or aberrant mTOR signaling pathway, may compromise megakaryocyte differentiation, producing negative impact on platelet production and function finally [20]. Besides, the Bcl-2 protein family is a group of highly conserved proteins with effects of regulating mitochondrial membrane permeability, cytochrome c release, and caspase activation, which has been extensively studied in megakaryocyte apoptosis [38]. As illustrated in Fig. 2, these mediators amplify platelet activation through specific receptor-ligand interactions, such as ADP binding to purinergic receptor P2Y1, TXA₂ to the thromboxane receptor (TP), and thrombin to PARs, leading to cytoskeletal reorganization and subsequent granule release.

## Understanding ITP from the perspective of TCM theory

ITP, with insufficient understanding of its etiology, falls within the category of “blood syndrome “ and “purpura” in the field of TCM. Dysfunction of the spleen to control blood”, “blockage due to blood stasis”, and “compromised blood circulation owing to pathogenic heat” are examples of relevant theories explaining its pathogenic mechanisms [39]. On the basis of the academic idea of “Yang transforming Qi and Yin constituting the body” in TCM theory, “platelet production” falls within the category of Yin essence transforming into blood, which is related to the generation of tangible substances; while “platelet destruction” belongs to the category of Yang Qi accelerating the decomposition of tangible substances. Despite no direct scientific concept of “Qi” in Western medicine, Zehnder et al. [40] reported that decitabine, a hypomethylating agent, can redefine Qi by restoring the balance of energy metabolism through the LKB1/AMPK pathway.

As stated in the “*Plain Questions (Su Wen): The Great Treatise on Tianyuan Ji*”, “change comes into being in the case of imbalanced Yin and Yang.” Given the production of platelets falling within the category of transforming Yin essence into blood mentioned earlier, the pathological process of excessive platelet destruction is mutually related to the dissipation of Yang Qi or abnormal platelet function. The “*Jin Kui Gou Xuan—VolumeI—Fire*” also proposed that “Excess Qi is fire.” There will be excessive “Yang transforming Qi” effect if true Yin is disturbed by internal and external factors, and its ability to restrain Yang Qi weakens. Therefore, according to the TCM theory of “Yang transforming Qi and Yin constituting the body”, the development of ITP may stem from the imbalance between “body constitution” and “Qi transformation”, which is accompanied by thrombocytopenia [41].

Patients in the chronic stage may be exposed to external pathogenic factors, and are more prone to overwork or physical deficiency. According to TCM theories of “Qi being the governor of blood”, and “blood being the mother of Qi”[42, 43]. Qi and blood are inseparable and serve as the key foundations for each other to perform their physiological functions. Qi deficiency fails to control blood, leading to the symptom of bleeding; and it may weaken the effect of promoting blood circulation, causing blood stasis and resulting in bleeding [44]. On the basis of the academic idea of “Yang transforming Qi and Yin constituting the body”, the generation of platelets falls within the category of “Yin essence transforming blood”.

## The regulatory mechanism of TCM on immunity

### The inhibitory mechanism of TCM on the abnormal activation of B cells and antibody production

B cells are core components of the humoral immune system, which have been found to be implicated in the pathogenesis of ITP through positive or negative immune regulation. There may be significantly decreased proportion of naive B cells, but increased proportion of memory B cells in patients with active ITP[45]. Memory B cells can initiate a more rapid and intense immune response through this antigen, promoting the differentiation of CD4^+^ T cells and exerting a positive regulatory effect, thereby accelerating the production of platelet antibodies [46]. CD72, a common receptor for B cells, plays a bidirectional regulatory role in B-cell receptor (BCR) signal transduction to regulate B-cell activation. CD4^+^ T cells can induce memory B cells to express CD72 in vitro. Interleukin (IL)-10 and B-cell activating factor (BAFF) can further increase the expression of CD72 in CD19^+^CD27^+^ B cells [45], with the detection of obviously lower CD72 mRNA expression in patients with active ITP than that in those in remission. Enhanced BCR signaling can prompt B cells to produce autoreactive antibodies, thereby triggering the occurrence of ITP [47]. More recently, paeoniflorin-type compounds have been discovered to exert a down-regulatory effect on the BAFF signaling axis. CP-25, a paeoniflorin derivative, can suppress B-cell activation and attenuate humoral hyperactivity by inactivating the BAFF/BAFF-R-PI3K/AKT/mTOR pathway[48]. Berberine could inhibit the T follicular helper (Tfh) cell program and the expression of related cytokines, thereby indirectly reducing germinal center (GC) reactions and pathogenic B-cell differentiation [49]. Moreover, the triterpenoid triptolide could markedly suppress B-cell differentiation and antibody production in multiple disease models, and further restrict the excessive activation of GC by modulating the Tfr/Tfh balance [50]. Clinically, ITP patients were examined with significantly lower number of Bregs as well as levels of IL-10 and TGF-β than those in the normal population [51]. A reduction in the number of Bregs within the bone marrow would lower the levels of IL-10 and TGF-β, which might suppress the expression of TNF-α and the differentiation of Th cells and regulatory T cells (Tregs), ultimately compromising the immune regulatory function [52]. Besides, the anti-platelet autoantibodies produced by their abnormal functions could activate specific targets of platelet membrane proteins [53].

In another study on 40 chronic ITP (CITP) patients by Wu et al., a treatment group and a control group were randomly established and provided with prednisone treatment similarly. Meanwhile, the treatment group was treated with *Jianpi Yiqi Shexue decoction (JYSD)* granules for 21 days [54]. Consequently, both groups of patients were detected with significantly higher platelet counts after treatment than those before treatment. Moreover, in the control group, there was no significant change in the pre- and post-treatment proportion of CD19^+^CD27^+^ and CD19^+^CD27^−^ B cells; but in the treatment group, this proportion was remarkably reduced after treatment compared with that before treatment. Similarly, Wang et al. [55] reported that ITP patients with Qi deficiency syndrome would have lower number of Bregs compared with healthy blank controls, and the number of Bregs was negatively correlated with Qi deficiency syndrome score. Collectively, the pathogenesis of ITP (Qi deficiency syndrome) may be related to a reduction in the number of Bregs.

### Regulatory mechanism of TCM herbs in T-cell immune intolerance

T-cell-mediated immune disorders constitute one of the key mechanisms of ITP pathogenesis. Anti-platelet autoantibodies may not be detectable in all ITP patients, and T cells can initiate, proliferate and maintain anti-platelet autoimmunity, which has been recognized as a core factor in immune pathogenesis [56]. The imbalance of T-cell subsets is a core pathological feature. In ITP patients, there would be significantly increased proportions of Th1, Th2, Th17, Th22 and Tfh cells in the bone marrow, simultaneously increased levels of plasma IL-22, IL-17A and interferon-γ (IFN-γ), as well as decreased proportions of Tregs in both the bone marrow and peripheral blood [57]. There was also obviously increased expression of Bmi-1 in the peripheral blood of CD4^+^ T cells from ITP patients in the active stage [58].

In response to the aforementioned T-cell immune disorders, TCM functions significantly in regulating T lymphocyte subsets. Chen Dan et al. reported that obviously lower pre-treatment counts of CD3^+^, CD3^+^CD4^+^, and CD4^+^/CD8^+^ cells, as well as evidently elevated counts of CD3+ CD8+ cells in 69 ITP patients than those in 40 healthy controls [59]. Notably, corresponding treatment resulted in a correction in the abnormality of this subset, confirming an intimate association of T-cell subsets with the onset of ITP. Similarly, Zhou et al. [60] randomly divided 60 ITP patients with Yin deficiency and hyperactivity of fire into two groups, and provided both groups with 4 weeks of treatment using prednisolone. The treatment group was also supplemented with modified *Xigen powder*, enabling Yin nourishing, fire clearing, blood cooling and bleeding arresting. Three months of treatment led to significantly increased number of CD4^+^ cells, obviously decreased number of CD8^+^ cells, and elevated platelet count in the treatment group, suggesting the efficacy of this formula in regulating the quantity and function of CD4^+^ and CD8^+^ cells. In terms of CD4^+^ subgroup regulation, Li et al. [61] randomly grouped 122 ITP patients, with the adoption of prednisone (10–60 mg/d) combined with *Yiqi Ziyin Shengxue Decoction* in the treatment, and prednisone (the same dosing) alone in the control group. Consequently, the treatment was observed with more significantly decreased TCM syndrome score. In addition, with the enrollment and random grouping of 63 newly diagnosed ITP patients, Sun et al. [62] applied the following therapeutic protocols of prednisone alone in the control group, and *Qingre Xiaoban Decoction* + prednisone in the treatment group. After treatment, the treatment group showed increased number of CD4^+^CD25^+^CD127^low+^ Tregs, decreased number of CD19+B cells, and highly increased platelet count. Besides, *Trametes robiniophila Murr* aqueous extract has also been explored with the effect of modulating immune signaling in CD4^+^ T cells of ITP patients [63].

### Regulatory mechanism of the phagocytic function of macrophages in TCM

Macrophages can mediate the phagocytosis of platelets through Fcγ receptors and destroy platelets through endocytosis, serving as an important mediator in the pathogenesis of ITP as well. Meanwhile, as antigen-presenting cells, macrophages can also present platelet glycoproteins to T cells and activate B cells. Then, B cells produce antibodies that are essential in inducing ITP-acquired immune responses [64, 65]. In terms of the specific category of Fcγ receptors, the activating types include FcγRI (CD64), FcγRIIa (CD32a), FcγRIIc (CD32c), and FcγRIII (CD16a), whereas the inhibitory type is FcγRIIb (CD16b) [66]. In ITP patients, monocytes can increase the expression level of FcγRI, leading to enhanced phagocytic function by altering the ratio of FcγRIIa to FcγRIIb [67]. Critically, phagocytic dysfunction can amplify platelet destruction. TCM enables the regulation of the phagocytic function of macrophages through multiple mechanisms, thus exerting possible therapeutic effect in ITP patients. The specific mechanisms are: ① regulating macrophage polarization: some TCM herbs can promote macrophage polarization toward the M2 type and inhibit M1-type polarization; ② inhibiting the release of inflammatory factors: TCM can inhibit the secretion of inflammatory factors by macrophages (e.g., TNF-α and IL-1β) to alleviate inflammatory responses; ③ modulating the balance of immune cells: TCM can indirectly affect the phagocytic function of macrophages by regulating immune cell (e.g., T cells and B cells) functions; and ④ improving the bone marrow microenvironment: TCM can improve the bone marrow microenvironment, promote the proliferation and differentiation of megakaryocytes, and increase platelet production, thereby reducing the clearance of platelets by macrophages. Collectively, TCM can regulate the phagocytic function of macrophages through multiple pathways, modulate immune balance, and improve the symptoms of thrombocytopenia in patients with ITP.

## Research on the mechanism of single active ingredients in TCM

**Fig. 3:**
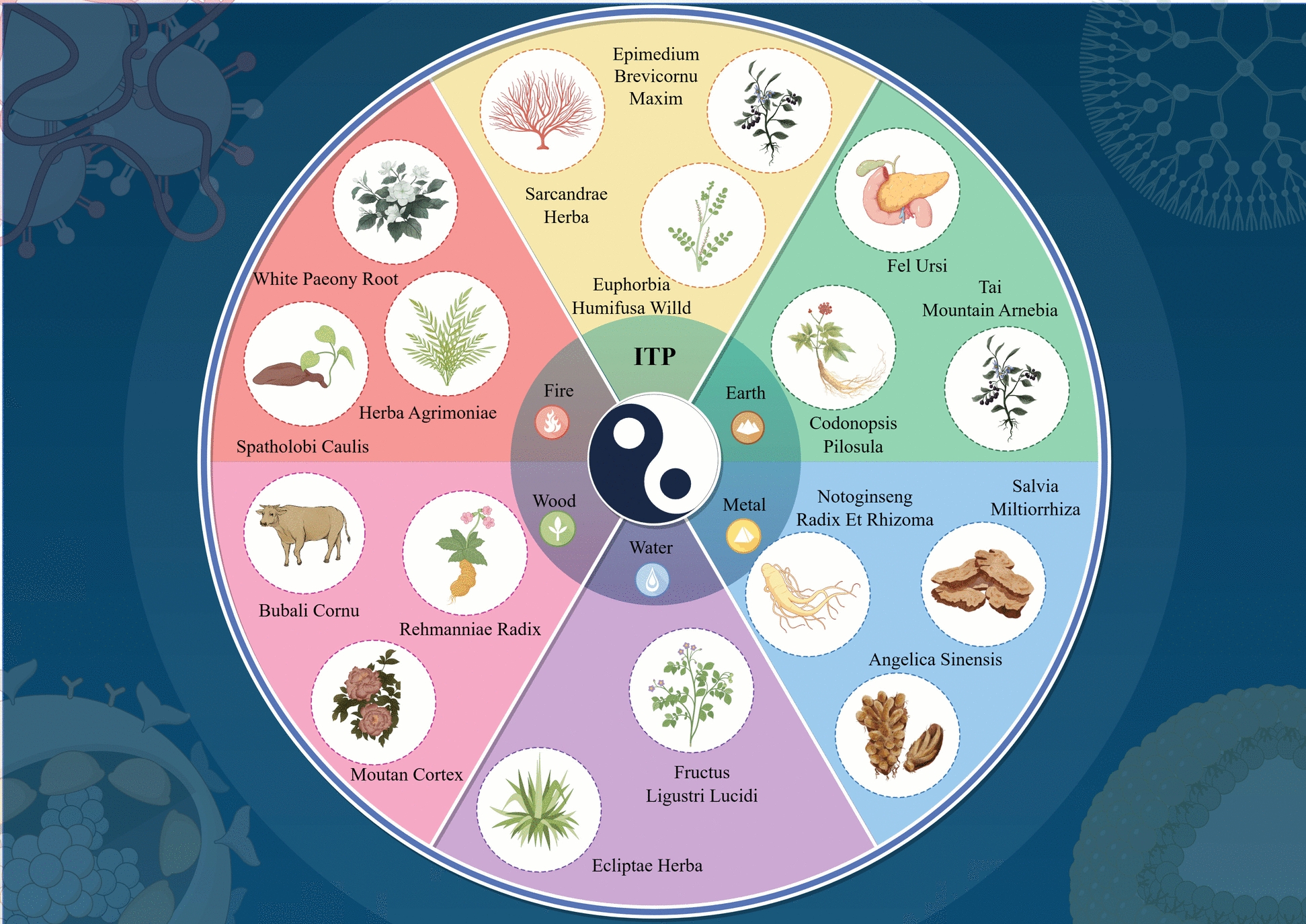
Research on the mechanisms of single active ingredients in TCM for ITP treatment. The diagram is organized around a Yin-Yang motif, symbolizing the dynamic balance of physiological processes. Each of the five segments corresponds to one of the Five Elements (i.e., Metal, Water, Wood, Fire, and Earth), and each links to specific TCM herbs and their proposed roles in modulating immune and hematopoietic functions in ITP

Accumulating evidence of research in recent decades have unveiled potential molecular mechanisms of single active ingredients in TCM for ITP treatment. Figure 3 provides a comprehensive overview of the principal pathways (e.g., immune modulation and hematopoietic promotion) underlying the therapeutic potential of these compounds. For instance, icaritin, an active component in Epimedium extract, can promote platelet production and regulate T-cell polarization, yet without clear definition of its underlying mechanism [68]. Certain extracts of TCM can also combat thrombocytopenia by improving the morphology and function of megakaryocytes [69]. Furthermore, network pharmacology has been widely applied in identifying active components of TCM, such as screening compounds with potential therapeutic effects through the TCM Systems Pharmacology Database [70–72] (Table 2). In order to facilitate a structured understanding of their roles in ITP management, Table 2 categorizes studies on single TCM active ingredients, detailing their efficacy, mechanisms, and experimental conclusions. Notably, priority should be given to conducting prospective, randomized, double-blind, placebo-controlled trials focusing on high-potential TCM compounds (e.g., *icaritin*) or formulations (e.g., *Guipi Decoction*), thereby accelerating the translation of these findings into clinical practice. In addition, these studies should employ consistent, predefined outcome measures, including platelet counts, hemorrhage grading scales, and corticosteroid tapering schedules.

**Table 2:** Study on the mechanism of action of the active ingredients of a single Chinese medicine

Name of TCM	Main active ingredients	Efficacy of TCM	Mechanisms in treatment of ITP	Relevant experimental/research conclusions
*White Peony Root*	Active components of raw paeony, active components of bran fried Paeony, and total glucosides of paeony	Nourish blood andregulate menstruation, astringe Yin andstop sweating, soften liver andrelieve pain, and calm liver Yang	Regulate platelet indexes: Increase platelet count and thrombocytocrit, decrease mean platelet volume; and platelet distribution width; Regulate Th1/Th2 balance: Increase Th1 factor (IFN-γ); and decrease Th2 factor (IL-4); Regulate immune function: Increase the proportion of CD4⁺CD25⁺Foxp3⁺Treg cells and Foxp3 mRNA expression, and enhance immunosuppressive function	In ITP mice, bran-fried peony exhibited a stronger effect than raw peony; ELISA results showed that bran-fried peony could significantly regulate IFN-γ increase and IL-4 decrease
*Crane herb*	Flavonoids, quercetin, Kaempferol, luteolin, and catechin	Astringe hemostasis, and tonify deficiency	Activate hematopoietic function: Promote proliferation and differentiation of hematopoietic and erythroid progenitor cells, and increase platelet count Regulate hematopoietic-related genes: Regulate erythropoietin receptor (EPOR) and key transcription factors (MYB, and PU.1); Key target genes: TNF, IL-6, IL-1β, STAT3, CCL2, CXCL8, and JUN; Regulate signaling pathway: Inhibit STAT3 activity to prevent Th17 cells; and activate PI3K-Akt pathway to promote blood cell regeneration	Combined use of Jixueteng was effective in treating ITP; Catechin could directly promote hematopoiesis-related processes Flavonoids could increase platelet count, but their effect in treating ITP required further verification
*Chicken blood vine*	Quercetin, kaempferol, luteolin, and catechin	Invigorate blood, clear heat, and detoxify	Activate hematopoietic function: Increase platelet count; Synergistic effect: Act together with the active ingredients of Crane grass on hematopoietic and immune-related targets to enhance the curative effect	Its active ingredients could activate hematopoietic function when used in combination with Crane herb. There were 29 active ingredients and 663 targets in total
*Codonopsis*	Codonopsis ginseng polysaccharide, glycitein, ethyl α-d fructofurantoside, and myristic acid	Tonify Qi, invigorate the spleen, benefit the lung, nourish blood, and promote body fluid	Regulate immune-related pathways: PI3K/Akt and NF-κB pathways; Regulate Treg/Th17 balance via PI3K/Akt pathway and IL2 targets; Core targets: IL2, and VEGFA, regulate cell differentiation, protein phosphorylation (PTP), participate in inflammatory response, angiogenesis, and remodeling	*Codonopsis ginseng* polysaccharide exhibited an immunomodulatory effect. Its main components could alleviate the symptoms of ITP by inhibiting the NF-κB pathway and regulating the PI3K/Akt pathway
*Tai Mountain Arnebia*	–	Invigorate blood, cool blood, clear heat, and detoxify	Increase the number of platelets: Reduce the degree of compensatory platelet proliferation; Regulate cytokines: Decrease IL-2 content, and increase IL-10 content; Regulate TPO level: Reduce serum TPO concentration, promote TPO uptake, utilization, and degradation	The efficacy of *Tai Mountain Arnebia* Decoction was similar to that of prednisolone acetate in the ITP mice, coupled with better regulatory effect on IL-10 It could reduce immune damage to platelets and increase peripheral blood platelet count
*Bear bile powder*	–	Close the vascular opening, stop bleeding, remove the "day of hope", brighten the eyes, embalm, and alleviate diarrhea	Modulate monocyte subsets: Inhibit migration of Ly6Chi pro-inflammatory monocytes from BM to circulation; Reduce inflammatory factors: Decrease the content of inflammatory factors (TNF-α, and IL-1β) in peripheral blood; Alleviate platelet immune destruction: Improve inflammatory response, and reduce T and B cell immune response triggering	Mongolian TCM prescription "Tonglaga-601" was effective in treating ITP; Intervention in ITP mice showed increased Ly6Chi monocytes in bone marrow, decreased Ly6Chi monocytes in circulation, and reduced inflammatory cytokines
*Brocade grass*	–	Clear heat and detoxify, cool blood and stop bleeding, dampness and yellow	Improve platelet function: Increase platelet count; Regulate T lymphocyte subsets: Increase CD3⁺, CD4⁺ and CD4/CD8 levels to correct the disorder of lymphocyte expression; Regulate Th1/Th2 balance: Reduce Th1 cytokines (IL-2, IFN-γ), increase Th2 cytokines (IL-4, IL-10), and decrease IFN-γ/IL-4 ratio	It was used to treat gingival bleeding and subcutaneous purpura; In treating ITP patients, it could correct T lymphocyte subsets and Th1/Th2 imbalance
*Sarcandra glabra*	Total flavonoids of Sarcandra glabra, Icaritin, phenols, tannin, coumarin, and lactone	Clear heat and cool blood, activate blood and eliminate spots, dispel pathogenic wind and dredge collaterals	Promote megakaryocyte function: Boost the proliferation; differentiation of bone marrow megakaryocytes; and improve bone marrow cells; Activate TPO-C-mpl pathway: Pormote megakaryocyte proliferation and differentiation to form platelets; Regulate immune balance: Reduce Th1/Th17 related cytokines, increase Th2/TGF-β, and regulate Th/CTL imbalance; Regulate signaling pathway: Down-regulate the expression of p-STAT3 in JAK2/STAT3 pathway to regulate bone marrow TPO/MPL metabolism	Combination with low-dose prednisone could increase PLT, CD3⁺, CD3⁺CD4⁺, CD3⁺CD16⁺CD56⁺ levels, and reduce the side effects of prednisone; Icaritin could increase PLT level, alleviate splenomegaly, and down-regulate TPO, MPL, and p-STAT3 expression in the bone marrow of ITP mice
*Tripterygium wilfordii*	Tripterygium wilfordii polyglycosides (diterpenoids and alkaloids)	Dispel wind dampness, promote blood circulation, relieve swelling and pain, kill insects, and detoxify	Regulate immune function: Increase CD4⁺/CD8⁺ ratio and CD4⁺CD25⁺T cells; Regulate metabolic pathways: Act via IDO/TTS-mediated tryptophan metabolic pathway	It could improve PLT count in elderly patients with recurrent ITP; and its efficacy was similar to dexamethasone, with mild adverse reactions
*Green flower*	Indirubin, and indigo	Clear heat and detoxify, cool blood and stop bleeding,, clear the liver and reduce pathogenic heat	Regulate thrombopoiesis-related genes: Enhance MPL expression; and inhibit TNF expression; Regulate immune function: Increase the number and function of Treg cells; and inhibit effector T cells; Regulate signaling pathway: Restore the expression of PD1 and PTEN, and increase the AKT/mTOR signaling pathway in CD4⁺T cells from ITP patients; Increase the number and percentage of CD4⁺CD25⁺Foxp3⁺Treg cells	Indirubin could enhance MPL and normalize TNF in PBMCs of ITP patients in vitro; In the chronic ITP mouse model, indirubin could significantly increase PLT levels and improve immune imbalance
*Burdock seed*	Arctigenin, lignans, fatty acids, and volatile oils	Evacuate pathogenic wind and heat, relieve sore throat and detoxify	Regulate immune function: Increase Treg cell ratio	Arctigenin revealed a good therapeutic effect on ITP animal models, and its mechanism was related to the regulation of Treg cells

### Herbs primarily promoting megakaryocyte maturation and platelet production

#### Epimedium

A number of TCM herbs have demonstrated significant potential in enhancing megakaryocyte proliferation, differentiation, and maturation, thereby elevating platelet counts by modulating key signaling pathways such as TPO/c-Mpl, PI3K/Akt, and JAK2/signal transducer and activator of transcription 3 (STAT3). For instance, *Epimedium brevicornum* Maxim., also known as the “*Epimedium* Herb”, is a perennial herb of the Berberidaceae family. It has a pungent and sweet taste, is warm in nature, and acts on the liver and kidney meridians. In murine models, the representative flavonoid monomers *Epimedium* and its deglycosylated derivative *icaritin* could significantly increase serum TPO levels, elevate peripheral platelet counts, and enhance platelet hematocrit [68]. Wang et al. [73] reported that *Epimedium* can regulate multiple signaling pathways by acting on key targets (e.g., UCHL5, TNFAIP8, and Visfatin), and can activate both the IRS1/Akt/mTOR/GSK3β pathway and the cell cycle pathway. It can also promote the differentiation of megakaryocytes by inhibiting the Hippo, JAK/STAT, NF-κB and other pathways. Initially, *Epimedium* polysaccharides were confirmed to restore Th17/Treg ratio and increase bone marrow nucleated cell count to exert therapeutic effects in aplastic anemia. This is believed to be mechanistically associated with inhibited expression of STAT3/RORγt and promoted expression of STAT5/Foxp3 [74, 75]. Sun et al. reported that *Epimedium* could significantly increase the PLT level in peripheral blood from ITP mice [76]. Immune cell regulation can improve the imbalance of Th/CTL cells by decreasing the number of Th1, Th17 and Tfh cells in the spleen, and increasing the number of Th2 and Tregs. Zhang et al. demonstrated that *Epimedium* could significantly increase the platelet count and improve the platelet aggregation ability in the peripheral blood, and also escalate the level of TPO in the serum of ITP mice [68]. Altogether, *Epimedium* may exert therapeutic potential in mice with ITP by downregulating the expression of p-STAT3 in the JAK2/STAT3 signaling pathway, and regulating the TPO/MPL metabolic process in the bone marrow.

#### Sarcandrae Herba

*Sarcandrae Herba* is a species of *Sarcandra glabra (Thunb.)* from the family Chloranthaceae. Flavonoids, phenolic acids, and coumarins are representative active components of *Sarcandrae Herba*. Modern reviews have demonstrated its comprehensive pharmacological profile, encompassing anti-inflammatory, antioxidant, and antithrombocytopenic activities. In murine models of ITP or thrombocytopenia, total flavonoids from *Sarcandrae Herba* could upregulate the TPO-c-Mpl and SDF-1-CXCR4 axes within the bone marrow microenvironment, promote megakaryocyte differentiation and maturation, and increase peripheral platelet count [77]. Zhu et al. [78] were the first to decipher the anti-purpuric effects of *Sarcandrae Herba*. They discovered that its total flavonoid components can inhibit mitochondria pathway-mediated platelet apoptosis, reduce phosphatidylserine exposure, decrease PAC-1 and P-selectin expression, prolong platelet survival, and increase platelet count. Moreover, the total flavonoids or flavonoid fractions derived from *Sarcandra glabra* could also inhibit excessive platelet apoptosis mediated by the mitochondrial pathway [78]. In addition, the total flavonoids of cytarabine could significantly increase the number of platelets in the peripheral blood of animals with cytarabine-induced thrombocytopenia. Its possible mechanism may be related to the activation of the TPO-C-mpl pathway to promote megakaryocyte proliferation and differentiation, thereby forming platelets.

#### Bear bile powder

*Bear bile powder,* a Mongolian medicine, is the dried gallbladder of black bears and brown bears used for clearing heat and detoxifying[79], which is cold in nature, bitter in taste, and acts on the liver and gallbladder meridians. The main active components of *Bear bile powder* are bile acids, particularly ursodeoxycholic acid (UDCA). Rcently, 24-norUDCA, the UDCA analogue, has been discovered to suppress pathogenic Th17 cells, promote Treg differentiation, and alleviate immune-related inflammation in various murine models [80]. Early studies have also confirmed the therapeutic effects of *bear bile powder* and ursodeoxycholic acid on Ara-C-induced thrombocytopenia model [81]. Intervention using both substances could increase platelet and megakaryocyte counts, which might be potential therapeutic agents for ITP. Moreover, *bear bile powder* may also exert a role in alleviating lipid metabolism disorders [82], which has been confirmed as one of the pathogenesis of ITP. For example, Chen et al. [83] demonstrated that the Mongolian medicine formula “Tonggelaga 601”, formulated with bile as the core component, can regulate the differentiation and maturation process of bone marrow megakaryocytes, in addition to effectively increasing platelet count, as well as exerting hemostatic and stasis-resolving effects, thereby exhibiting significant therapeutic effects on ITP treatment.

#### Spatholobus suberectus

According to modern pharmacological research, *Spatholobus suberectus* can promote the regeneration of blood cells and increase the number of platelets through the synergy of multiple components, targets and pathways [84]. For patients diagnosed as ITP categorizing into the TCM syndrome of liver depression with severe blood deficiency, *Spatholobus suberectus* is often used in combination with *Angelica sinensis* to promote blood circulation and replenish blood. Moreover, the addition of *Cymbidium goeringii* and *Spatholobus suberectus* in TCM prescriptions may exert preferable effect on ITP. Modern pharmacological studies also show that *Agrimonia pilosa* and *Spatholobus suberectus* have multiple effects, such as activating hematopoietic function and increasing the platelet count. *Spatholobus suberectus* contains 29 active ingredients and 663 targets. Quercetin, kaempferol, luteolin and catechins can all activate hematopoietic functions and increase the platelet count [85]. In the treatment of ITP, key target genes of *Spatholobus suberectus* primarily acts on key target genes such as tumor necrosis factor (TNF), IL-6, IL-1β, STAT3, chemokine ligand 2 (CCL2), CXC ligand 8 (CXCL8, also known as IL-8), and chromosome 1 genes [86]. Noticeably, *Spatholobus suberectus* extract exhibits well-defined antiplatelet aggregation activity in both in vitro and animal studies. However, this antiplatelet pharmacological property may exacerbate bleeding risk in patients with ITP, thus warranting cautious clinical application [87].

#### Raw rehmannia

*Raw rehmannia* has a sweet and bitter taste and a cold nature. It is commonly applied for treating diseases caused by the invasion of pathogenic heat due to epidemics, as well as various blood-heat-induced bleeding symptoms [88], such as hematemesis, epistaxis, hematuria, and metrorrhagia. As an active component of *Rehmannia glutinosa*, catalpol is capable of promoting blood circulation and removing blood stasis through the downregulation of PI3K and AKT activities possibly. In addition, *Rehmannia polysaccharides* can promote bone marrow hematopoietic stem cell proliferation and differentiation, and can effectively increase platelet count [89].

#### Notoginseng

*Notoginseng*, with a sweet and slightly bitter taste and a warm nature, mainly acts on the liver and stomach meridians. It has an extensive application in the control of various symptoms, including hemoptysis, hematemesis, epistaxis, hematochezia and internal bleeding problems (e.g., metrorrhagia and menorrhagia) [90]. Its main active ingredient, *otoginsenoside*, can increase platelet count and shorten the coagulation time, prothrombin time and thrombin time [91]. Furthermore, the total saponins of *Panax Notoginseng* leaves, as the core component of exerting their blood-activating and stasis-resolving effects, can effectively inhibit platelet aggregation to function in preventing thrombosis. *Notoginseng* saponins and amino acid derivatives such as dencichine (aspartic acid derivative) can regulate the development of thrombopoiesis by boosting megakaryocyte adhesion, migration, and proplatelet formation [92]. Yang et al. found that four Ginsenoside subtypes in *Notoginseng* can regulate T-cell subset balance and B-cell-related antibody production, thus revealing the mechanism for treating ITP [93]. Furthermore, fermented *Notoginseng* saponins also possess higher bioavailability [94]. Animal research has documented the effects of total and fermented *Notoginseng* saponins in enhancing peripheral platelet, erythroid, and leukocyte counts, as well as hematopoietic factor levels, in bone marrow suppression models, suggesting their beneficial effects on hematopoietic recovery [94]. Early in vitro experiments comparing notoginsenoside R1 with recombinant thrombopoietin (TPO) also provided preliminary evidence supporting its thrombopoietic potential [95]. In addition, a latest review in 2025 comprehensively summarized the pharmacological activities of saponins from *Panax Notoginseng* [96].

#### Codonopsis pilosula

*Codonopsis pilosula*, an herb of the *Campanulaceae* family, has a rhizome and is a commonly used medicinal material in TCM. It is sweet, neutral in nature, and acts on the spleen and lung meridians, with functions of strengthening the spleen and lungs, nourishing blood and promoting body fluid production [97]. It contains various chemical components that can regulate human immune function, such as sugars (e.g., *Codonopsis pilosula* polysaccharides, CPPs), alkaloids and flavonoids [98, 99]. Among these, CPPs can regulate the immunity and promote gastrointestinal function, which are generally used jointly with *Astragalus membranaceus*, *Radix liquiritiae*, etc. [98]. Recent systematic reviews have revealed that CPPs can modulate innate immune pathways, notably the TLR4/MyD88/NF-κB axis, and coordinately regulate T cells, B cells, and NK cells. CPPs also possess functions of bidirectional modulation of cytokines, complement activity, and immune organ indices, thus exhibiting the characteristic “Qi-tonifying and immune homeostasis-maintaining” profile [100]. A retrospective review in 2024 systematically summarized the biological activities and pharmacological effects of *Codonopsis pilosula* [97]. The active components of *Codonopsis pilosula*, such as soybean flavin and ethyl α-D-fructose furanoside, can regulate Treg/Th17 cells through the IL-2 target of the PI3K/Akt pathway and inhibit the NF-κB pathway, ultimately alleviating the symptoms of ITP. Furthermore, numerous recent studies have confirmed an effective management of ITP, refractory ITP (RITP) in particular, by TCM compounds or mixtures containing *Codonopsis pilosula* [101]. Zhang et al. reported the application of modified *Sijunzi decoction* containing *Codonopsis pilosula* at different doses in ITP mice, which could increase multiple indicators, such as platelet count and thrombopoietic hormone [102]. Similarly, modified *Sijunzi granules* could mitigate the symptom of bleeding by activating the MAPK/Erk and PI3K/Akt pathways [103]. Zheng et al. demonstrated that *Guipi decoction* with *Codonopsis pilosula* combined with dexamethasone could effectively improve the clinical progression of ITP (Qi-deficiency type) [104]. In addition, TCM prescriptions containing *Codonopsis pilosula* were also discovered to have satisfactory preventive and therapeutic effects on ITP mice or patients in animal experiments or clinical trials [62, 105].

### Herbs primarily regulating T-cell immunity

Several TCM herbs can alleviate abnormal autoimmune-related platelet destruction in ITP. Its mechanism may be linked to the modulation of T-cell subsets, cytokine profiles, and immunosuppressive functions, such as elevating Tregs, rebalancing Th1/Th2/Th17 ratios, as well as inhibiting T- and B-cell proliferation.

#### White peony roots

*White peony roots*, which have a bitter, acidic and slightly cold taste, mainly exert pharmacological effects on the liver and spleen meridians [106]. Its main active constituents, *paeoniflorin* and *total glucosides of paeony*, exert immunomodulatory and anti-inflammatory effects. These effects are mediated by inactivating the NF-κB/MAPK, PI3K-AKT-mTOR, and JAK2-STAT3 pathways, as well as by modulating B cells, T cells, and dendritic cell responses, leading to an improvement in the autoimmune inflammatory microenvironment eventually [107, 108]. Notably, *Ejiao Siwu Decoction* containing the components of *white peony* has been shown to alleviate inflammation and promote platelet homeostasis by targeting core inflammatory nodes such as AKT1, TNF, IL-6, and CASP3 [109]. *Paeoniflorin* can also selectively inhibit shear-induced platelet aggregation, which, however, was recommended to be used with caution in ITP patients due to potential bleeding risks, although it did not prolong the bleeding time in animal models [110]. Clinically, *white peony root* is also a frequent therapeutic option given its ability to effectively regulate multiple key links in the autoimmune process [111, 112].

#### Tripterygium wilfordii

*Tripterygium wilfordii Hook.f.* belongs to the genus *Tripterygium* of the Euphorbiaceae family. The medicinal part is its dried root or the xylem of the root. It is bitter, cold and pungent in taste, and acts on the liver and kidney meridians; moreover, it is highly toxic and has significant medicinal effects [113]. From the perspective of chemical composition, *Tripterygium wilfordii* contains various active substances (e.g., alkaloids, diterpenoids, triterpenoids, sesquiterpenoids, glycosides, etc.), among which diterpenoids and alkaloids are the core components that exert the main pharmacological activities. Modern research has confirmed its multiple effects, such as immune regulation, antitumor, microcirculation improvement, anti-inflammation, as well as bactericidal and antipyretic analgesic effects [114]. Clinically, *Tripterygium* glycoside preparations combined with recombinant IL-11 was proven to improve certain clinical outcome measures in ITP patients [115]. Jiang et al. reported that treatment with *Tripterygium glycoside* tablets for elderly patients with recurrent ITP could increase the ratio of CD4^+^/CD8^+^, the level of CD4^+^CD25^+^ T cells, and the count of PLT, with mild degree of adverse reactions [116]. Moreover, Han et al. revealed that cyclosporine A combined with *tripterygium* glycosides could achieve a total effective rate of 76.69% in the clinical treatment for patients with RITP, accompanied by significantly greater post-treatment platelet count than that before treatment [117].

#### Indigo

*Indigo naturalis* possesses a diverse raw material sources, which originate mainly from the leaves or stems of three types of plants. Indirubin and indigo are its main active ingredients, among which indirubin is the main raw material of the TCM compound *Huangdai* [118]. Modern pharmacological studies have documented the anti-inflammatory, immunomodulatory, antibacterial, antiviral, antitumor and analgesic effects of *Indigo naturalis* [119, 120]. Shao et al. treated ITP patients and healthy individuals with indirubin at different concentrations, followed by the isolation of peripheral blood mononuclear cells (PBMCs) for in vitro experiments to observe its effect on the expression of PBMC-related genes [121]. In the CITP mouse model, indirubin significantly increased the count of PLT. Corresponding results supported that indirubin might exert therapeutic effects on ITP by regulating the homeostasis of the PD-1/PTEN/AKT signaling pathway in CD4^+^ T cells. Furthermore, Zhao et al. confirmed that *Indirubin* could significantly increase the quantity and percentage of CD4^+^CD25^+^Foxp3^+^ Tregs in the peripheral blood, spleen, thymus and lymph nodes of CITP mice [122], which was consistent with the earlier results reported by Zhang et al. [123] These findings further indicate that increasing the number of CD4^+^CD25^+^Foxp3^+^ Tregs and maintaining their immunosuppressive function may be critical for *indirubin* in improving the pathological state of ITP.

#### Euphorbia humifusa Willd

*Euphorbia humifusa Willd*, also known as Milk Grass, is also a common TCM with mild nature and pungent taste, acting on the liver, stomach and large intestine meridians. It exhibits multiple functions, such as clearing heat and detoxifying, cooling blood and stopping bleeding, reducing jaundice, softening the liver and easing joint movement, promoting diuresis and reducing swelling[124]. According to the modern pharmacological research, *Euphorbia humifusa Willd* contains various active chemical components, such as flavonoids [125], tannins, and alkaloids. Given the interaction of these components, this TCM possesses multiple biological activities, including anti-inflammatory, antibacterial, antioxidant, liver-protective, and cholagogic effects, providing a scientific basis for the modern interpretation of its traditional efficacy [126, 127]. Dong et al. [128] confirmed that *Euphorbia humifusa Willd* can significantly increase the number of platelets per unit volume and enhance platelet aggregation, strengthening its application in improving platelet-related indicators. Lu et al. [119] detected pre- and post-treatment T lymphocyte subsets in ITP patients via flow cytometry, revealing significantly larger number of CD3^+^CD4^+^ cells and greater CD4+/CD8+ T-cell ratio *Herba Euphorbiae Humifusae* Tablets-treated patients than those in the control. Therefore, *Euphorbia humifusa Willd* tablets can effectively correct lymphocyte disorder in patients with ITP. However, the latex and diterpenoids contained in *Euphorbia* species may irritate the skin and mucous membranes, and trigger gastrointestinal discomfort [129], highlighting the necessity for a comprehensive evaluation of the systemic safety of its long-term use.

#### Ligustrum lucidum

*Ligustrum lucidum*, with sweet and bitter taste, and cool nature, has medicinal effects on the liver and kidney meridians mainly. *Shennong’s Classic of Materia Medic*a classifies *Ligustrum lucidum* as a superior grade, stating that it mainly tonifies the middle-jiao and stabilizes the five internal organs [130]. *Ligustrum lucidum* is rich in triterpenoids (*oleanolic acid,* and *ursolic acid*), phenylethanoid glycosides (*acteoside/verbascoside*), and secoiridoid glycosides (*ligustroside,* and *nuezhenide*). It can exert anti-inflammatory, antioxidant, and immunomodulatory activities by modulating key pathways such as NF-κB, MAPK, JAK/STAT, Nrf2/HO-1, and the NLRP3 inflammasome [131, 132]. Modern research indicates that *Eclipta prostrata* has pharmacological activities, such as increasing the platelet count, shortening the prothrombin time, and regulating the immune function [133]. In the investigation of the effect of ethanol and ethyl acetate extracts from the whole plant of *Eclipta prostrata*, Guo et al. reported that these extracts could significantly accelerate the aggregation of red blood cells into clusters and form a reticular structure [134]. *Ligustrum lucidum* can tonify the liver and kidneys, and clear pathogenic heat. Preclinical and combination therapy research has revealed the roles of formulations containing *Ligustrum lucidum* in promoting hematopoietic recovery and ameliorating cytopenia in cyclophosphamide-induced bone marrow suppression models [135]. The auxiliary treatment of ITP primarily involves the syndrome of Yin deficiency of the liver and kidney. The combination of the two *Eclipta prostrata* and *Ligustrum lucidum* can enhance the effect of nourishing Yin and stopping bleeding, which, however, is suitable for ITP patients with the syndrome of Yin deficiency of the liver and kidney. If it is a syndrome of blood heat with erratic bleeding or Qi deficiency and blood stasis, this therapy may not be applicable and may even aggravate the condition.

#### Tai Mountain Arnebia

As one of the four famous medicines of Mount Tai, *Tai Mountain Arnebia* is a perennial herbaceous plant of the Boraginaceae family. In TCM, it can clear heat and cool blood, promote blood circulation and detoxify, dispel rashes and remove spots [136]. In general, the core bioactive constituents of *Arnebia* and *Lithospermum* species are naphthoquinones, which have been systematically identified, with wide distribution across both genera [137]. In terms of immunoregulation, these compounds can upregulate CD4⁺Foxp3⁺ Tregs and prolong graft survival in allogeneic transplantation models, underscoring their effects of inducing peripheral immune tolerance and restoring immune homeostasis in ITP [138]. As for their regulatory role in inflammatory signaling, they can inhibit the NF-κB/MAPK pathways and the NLRP3 inflammasome, which are also key mediators of immune dysregulation in ITP [139]. In a mouse ITP model with the administration of Taishan Zicao decoction via gavage, Yin et al. [140] discovered that this prescription could significantly increase the number of platelets in the peripheral blood of the modeled mice, effectively improve thrombocytopenia, and reduce the degree of compensatory proliferation of platelets. Notably, thrombopoietin (TPO) is a key indicator that regulates platelet count and is closely related to anti-platelet antibodies. Yin et al. also noticed a significantly lower TPO level in the *Tai Mountain Arnebia* decoction-treated group than that in the control [141].

### Herbs with hemostatic and blood-cooling properties

A subset of TCM herbs primarily exerts hemostatic effects by cooling blood, clearing heat, and modulating coagulation or antibody production. As a result, these herbs are highly suitable for ITP with manifestations of blood heat and massive hemorrhage.

#### Cranes Herb

*Cranes Herb* (*Agrimonia pilosa*), the dried aerial part of *Lysimachia glabra* (Rosaceae), is neutral, bitter, and astringent, and acts on the heart and liver meridians. It promotes astringency, stops bleeding, treats malaria and dysentery, detoxifies, and tonifies deficiency. With active ingredients that regulate intrinsic and extrinsic coagulation pathways, it possesses a hemostatic effect that can increase platelet counts. For instance, galactose and esterified rhamnuronic acid can inhibit plasma coagulation in vitro. Known as "Tuo Li Cao" in the *Diannan Materia Medica Atlas*, it is indicated in *Modern Practical Chinese Medicine* for anemia, weakness, and mental fatigue. For ITP with the syndrome of spleen deficiency-induced purpura, the addition of 10–30 g of *Cyperus nutans* can exert good therapeutic effect by strengthening the spleen and tonifying Qi.

#### Buffalo horn

The *buffalo horn*, which has a bitter taste and cold nature, acts mainly on the heart and liver meridians. Clinically, *buffalo horns* are often used to treat symptoms caused by the invasion of pathogenic heat into the blood due to typhoid or warm epidemics. Meanwhile, *red peony root*, which has a bitter taste and a slightly cold nature, mainly exerts its medicinal effect on the liver meridian. Modern pharmacological research has revealed that *red peony root* has immune regulatory effect. Given its taste and nature, peony bark functions to clear heat and cool blood, promote blood circulation and remove blood stasis. In addition, *Paeonia rubra* can improve the permeability of capillaries and effectively inhibit the production of platelet antibodies. Systematic reviews of Moutan cortex (*Paeonia suffruticosa root bark*) have highlighted its anti-inflammatory, antioxidant, and immunomodulatory properties, supporting its role as a key “blood-cooling and stasis-resolving” component in herbal formulations developed for ITP treatment [142].The three herbs, *red peony root*, *peony bark* and *white peony root*, contain components such as paeoniflorin, benzoyl paeoniflorin and paeonol that can prevent platelet aggregation [143]. Medicinal materials such as *buffalo horns*, *raw rehmannia*, *peony bark* and *red peony roots* is a combined therapy formed by Professor Huiping Yu, a nationally renowned TCM doctor, for treating ITP caused by blood heat[144].

## Biological mechanism of TCM compound therapy for ITP

**Fig. 4:**
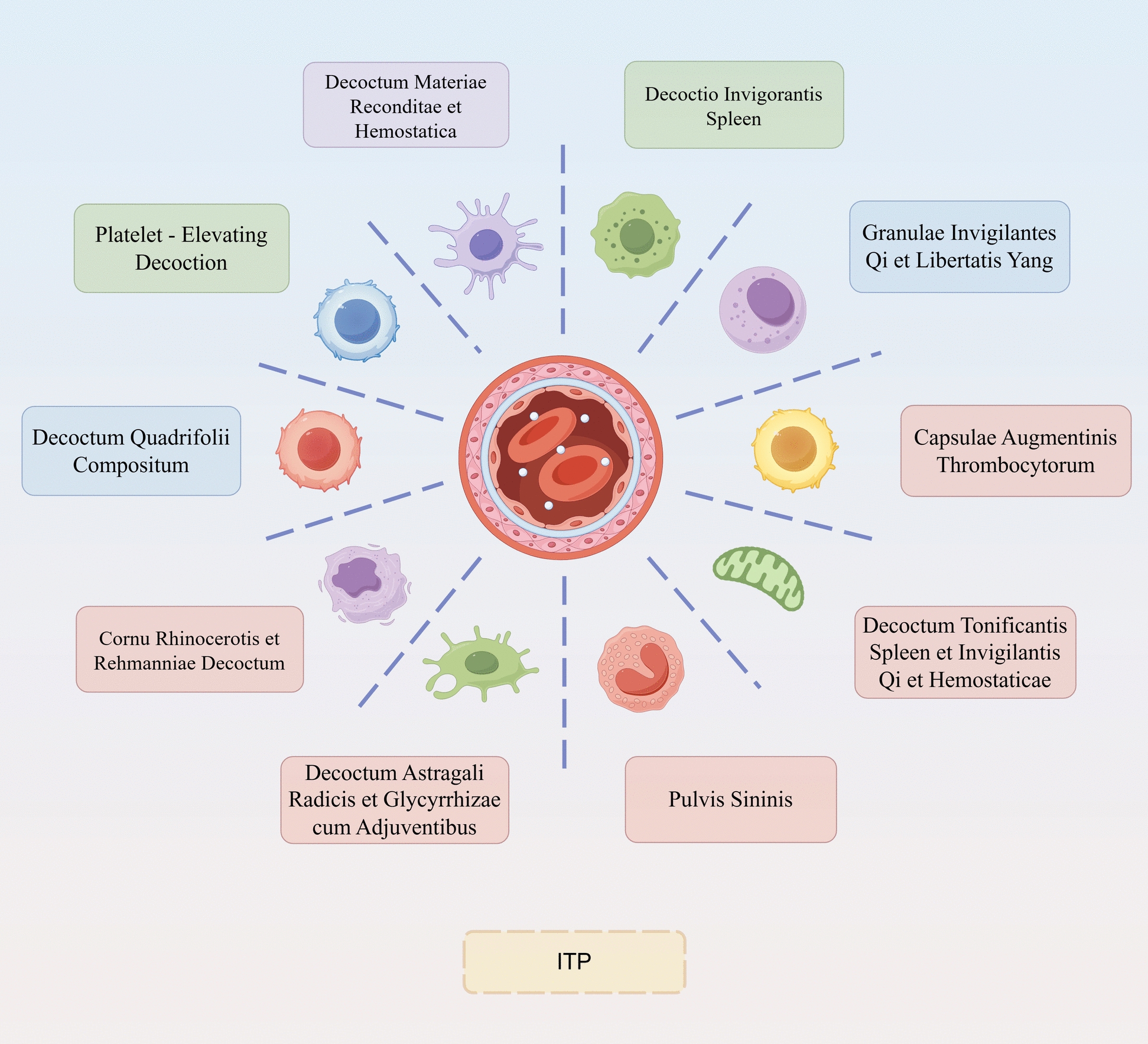
Research on the biological mechanisms of TCM compounds for ITP treatment. The schematic centers on ITP pathogenesis, illustrating the underlying mechanisms of various TCM herbal decoctions in exerting multi-target effects across key pathological pathways. Each formula is aligned with specific mechanisms, such as immunomodulation, promoted megakaryocyte maturation, or platelet production, highlighting systemic effects of TCM compounds in restoring homeostasis

For investigating compound research, network pharmacology is a common and valuable tool that has been widely applied to reveal the systemic biological mechanisms of TCM compounds for ITP treatment. As summarized visually in Fig. 4, a series of relevant studies have conducted to unveil the biological mechanisms of TCM compounds for treating ITP systemically, highlighting the involvement of multi-target interactions and pathway modulations. The *JYSD* Formula, a classic TCM compound, has been proven to exert a therapeutic effect on ITP by improving hematological parameters and coagulation indicators as well as promoting megakaryocyte proliferation and differentiation [145]. Subsequent studies further revealed that *JYSD* can regulate 5-hydroxytryptamine (5-HT) level in the blood of ITP patients through the gut-brain axis, thereby activating the coagulation mechanism [146]. *Yiqizi Yin* is another TCM formula developed based on the syndrome analysis of ITP in TCM [147]. It can modulate CD4⁺ T cell differentiation through the PI3K‒Akt signaling pathway, contributing to the restoration of immune balance in ITP patients eventually. Spleen-strengthening, kidney-nourishing and pathogenic fire-purging formulas contain multiple active ingredients targeting the regulation of M1-type macrophage polarization, yet with further clarification required to identify their key active ingredients and molecular targets [148]. For example, a prior research employed high-performance liquid chromatography‒mass spectrometry to identify active ingredients of *Ejiao Siwu decoction*, combined with a combined application of network pharmacology for clarifying its multitarget mechanism in the treatment of ITP [149]. These studies provide important evidence for understanding the systemic biological mechanism of TCM compounds for ITP treatment, and highlight the significance of quality control for the clinical application of TCM compounds (Table 3). Table 3 outlines the systematic biological mechanisms of representative TCM compounds in ITP treatment. Through the specification of their core efficacy, active ingredients, molecular targets, signaling pathways, underlying mechanisms, and experimental conclusions, we may acquire a structured comparative framework for their therapeutic actions.

**Table 3:** Systematic biological mechanism of TCM compounds in the treatment of ITP

TCM	Core efficacy and indications	Main active ingredients	Core action targets	Key signaling pathways	Core mechanisms for treating ITP	Relevant experimental/research conclusions
*Guipi Decoction*	Invigorate spleen/Qi, nourish blood/stop bleeding; For Qi deficiency or Qi deficiency without excess	Quercetin, kaempferol, isorhamnetin, β-sitosterol, formononetin, stigmasterol, calycosin, proopioid, and glycyrrhizin F	TNF, IL-6, IL-1β, IL-8, IL-10, IL-4, IL-2, PTGS2, IFNG, PPARG, HSP90AA1, CD206, CD80, CD86, IDO, an NF-κB p65	TNF, HIF-1, Toll-like receptor, T cellular receptor, and NOD-like receptor	Regulate immune function: Promote macrophage M2 polarization, increase Treg cells and TGF-β1, and inhibit T/B cell activation; Exert anti-inflammatory/anti-autoantibody effects: Inhibit NF-κB p65 phosphorylation, and reduce spleen CD80/CD86 and platelet autoantibodies; Regulate inflammatory factors	The combined use of prednisone and rhTPO (14-21d) achieved an efficacy of 88.75%; improved bleeding, fatigue; It also increased platelet count and inhibited B lymphocyte activation in ITP mice
*Sini San*	Soothe liver/regulate spleen, and balance Yin-Yang; For liver-spleen stagnation, and Yin-Yang disorder	Ganunolactone, licorice chalcone B, paeoniflorin, syringoid terpene, and tetramethoxyluteolin	TNF, VEGFA, STAT3, MMP9, CASP3, HRAS, PTGS2, TLR4, HIF1A, and IL-2	PI3K-Akt, cancer, AGE-RAGE, hepatitis B-related, and proteoglycan-cancer	Promote megakaryocyte maturation/thrombopoiesis: Accelerate Dami cell differentiation, and induce apoptosis to release platelets; -Improve hematopoietic microenvironment: Correct "marrow prosperity but blood failure", and promote bone marrow stem cell differentiation	Drug-containing serum inhibited Dami cell proliferation in a concentration–time dependent manner; It also improved megakaryocyte maturation disorder and increased platelet-forming megakaryocytes in ITP mice
*Xijiao Dihuang Decoction* (Including Combined Formula)	Clear heat/detoxify, and cool blood/stop bleeding; For blood-heat syndrome (combined formula nourishes Qi/Yin for essence deficiency)	Kaempferol, luteolin, quercetin, syringolipin diglucoside, β-sitosterol, isorhamnetin, and stigmasterol (combined formula: active ingredients of *Angelica sinensis*, *Astragalus membranaceus*, lotus, and *Ligustri lucidum*)	L-17, IL-35, Tregs/Th17 related targets, and inflammasome	Immunomodulation, and anti-inflammation	Regulate immune balance: Up-regulate Treg cells, balance Tregs/Th17, and adjust IL-17 (down) and IL-35 (up); Exert anti-inflammatory effect/protect vessels: Inhibit inflammasome activation, and reduce pro-inflammatory factors; Regulate platelet function: Flavonoids inhibit platelet aggregation; β-sitosterol/stigmasterol exert anti-inflammatory effect	It reduced ITP bleeding risk; could be used for severe emergency treatment; A combined use with Danggui Buxue Decoction and Erzhi Pillcould improve symptoms of ITP mice; and flavonoids' anti-platelet effect was correlated with B-ring phenolic hydroxyl number
*Jianpi Yiqi Yangxue Zhixue Fang*	Invigorate spleen/Qi, and nourish blood/stop bleeding; For Qi deficiency, Qi-blood deficiency	–	SDHA, ClpP, and LonP1	Mitochondrial energy metabolism (tricarboxylic acid cycle, and oxidative phosphorylation)	Maintain mitochondrial homeostasis: Regulate SDHA, improve complex II deficiency; Alleviate mitochondrial stress: Down-regulate abnormal ClpP/LonP1, and reduce oxidative stress; Improve hematopoietic energy supply: Restore mitochondrial ATP synthesis	It could increase platelets and improve bleeding in ITP mice; without dose dependence on SDHA/ClpP/LonP1 regulation; It could alleviate mitochondrial stress without hormone side effects
*Jianpi Zishen Xiehuo Decoction*	Invigorate spleen/kidney, and clear heat/reduce fire; For Yin deficiency with internal heat		IL-10, IFN-γ, TNF-α, IL-4, CD86, IL-12p70, and LMP2	JAK/STAT, and immune cell activation	Regulate Th1/Th2 balance: Down-regulate Th1/IFN-γ/TNF-α, and up-regulate Th2/IL-4/IL-10; Inhibit abnormal immune activation: Reduce splenic DC/CD86/IL-12p70, and block T cell activation; Regulate signaling pathway: Down-regulate LMP2, and inhibit JAK/STAT	A combined use with prednisone yielded an efficacy of 97.06%; and it could also assist hormone withdrawal, and improve symptoms; It could down-regulate LMP2mRNA in ITP patients' PBMC, balance Th1/Th2, and reduce platelet destruction
*Yangyin Jiangre Fang/Huoxue Fang*	Yangyin Jiangre Fang: Nourish Yin/clear heat; Huoxue Fang: Promote blood circulation/remove stasis; For heat forcing blood flow, and blood stasis	Yangyin Jiangre Fang: Active ingredients of ripe *Rehmannia glutinosa*, white peony root, *Eclipta herba*, and *Ligustri lucidum*; Huoxue Fang: Active ingredients of *Angelica sinensis*, *Salvia miltiorrhiza*, Jixueteng, and *Panax notoginseng*	Bcl-2, and Fas	Mitochondria-mediated apoptosis	Promote spleen lymphocyte apoptosis: Yangyin Jiangre Fang down-regulates Bcl-2; and Huoxue Fang up-regulates Fas; -Reduce autoantibodies: Shorten B cell lifespan via activated lymphocyte apoptosis	In ITP mice, it was observed with up-regulated spleen Bcl-2 and down-regulated Fas; Yangyin Jiangre Fang could down-regulate Bcl-2, Huoxue Fang could up-regulate Fas; -It could alleviate splenomegaly, and reduce platelet phagocytosis/destruction
*Yiqi Tongyang granules*	Tonify Qi/Yang, and increase platelets; For Qi-Yang deficiency	–	Bone marrow megakaryocyte differentiation-related targets, Th cells, Treg cells, B cells	Megakaryocyte differentiation/maturation, and immune subset regulation	Optimize megakaryocyte differentiation: Reduce primitive/naive megakaryocytes, and increase platelet-producing ones; -Correct immune subset imbalance: Increase Th/Treg percentages, and regulate T/B ratio; Protect immune organs: No thymic atrophy	It could significantly increase the PLT count in ITP mice; showing better efficacy than western medicine; It exhibited no thymus inhibition; and could avoid hormone-induced immune organ damage

### Compounds primarily promoting megakaryocyte maturation and platelet production

In this section, we attached our attention to TCM compounds with primary effects of enhancing megakaryocyte proliferation, differentiation, and maturation, thereby increasing platelet counts through mechanisms such as improving mitochondrial function, inducing polyploidization, and supporting hematopoiesis. These formulations address the root deficiencies of Qi and blood in ITP, and complement immune-modulating therapies by directly promoting platelet restoration.

#### Flavored Siwu Decoction

*Siwu decoction* is a classic blood-nourishing formula in TCM. The entire formula embodies the principles of combining dynamics and statics, as well as coupling hardness with softness, thereby achieving effects of nourishing blood without causing blood stasis and harmonizing blood without causing any damage. The modified *Siwu decoction* was derived from “Twenty Years of Fu Qingzhu's Gynecology”. Prior research has identified key therapeutic targets (i.e., AKT1, TNF, IL-6, CASP3, and TP53) of this compound in ITP by integrating HPLC-based qualitative and quantitative analyses with network pharmacology and molecular docking. It has been discovered that it might modulate the VEGF, AGE-RAGE, and complement-coagulation cascade pathways to downregulate TNF-α and IL-1β, enhance vascular integrity, and promote platelet recovery [150]. Feng et al. [151] demonstrated that modified *Siwu decoction* could increase the platelet count, and alleviate megakaryocyte-biased hematopoiesis, revealing good therapeutic effect on ITP. In addition, Feng et al. [152] also reported that modified *Siwu decoctio*n had the same therapeutic effect as prednisone did, manifesting as significant increase of the number of blood cells in the peripheral blood and obvious reduction of the content of platelet-associated antibodies in ITP mice.

#### Sini SAN

The *Sini SAN* is fundamental prescription originated from the classic work of TCM, “Treatise on Febrile Diseases”, holding an important position in the clinical system of TCM given its functions of soothing the liver and regulating the spleen. Professor Zeng Yingjian has provided unique insights into the diagnosis and treatment of ITP, suggesting that its pathogenesis has an intimate association with the functional disorders of two major organs, the liver and the spleen: the normal function of the spleen and stomach Qi transformation may be impaired in the context of the liver failing to regulate the flow of Qi [153]. On this basis, he took the *Sini SAN* as the basic prescription for treating ITP, adhering to the therapeutic principle of “regulating the Shaoyin meridian and opening the Taiyin meridian”, combined with a flexible adjustment according to the specific symptoms of specific patients. This treatment focuses on treating both manifestation and root cause of disease, which can improve the internal environment of the body by regulating the smooth flow of Qi and harmonizing Qi-blood circulation. Yu et al. [154] reported that *Sini san* could promote the differentiation and maturation of Dami cells by up-regulating the expression of key factors (e.g., Cyclin D3, GADA-1, and NF-E2), thereby inducing Dami cell polyploidization and providing more sources of mature megakaryocytes for platelet production.

#### Spleen-strengthening, Qi-Tonifying and blood-regulating formulas

The formula for strengthening the spleen, tonifying Qi and regulating blood is developed based on the classic prescription of *Sijunzi Decoction*. The addition of *Astragalus membranaceus*, *donkey-hide gelatin* and *Rubia milii* can further enhance the effect of consolidating Qi and preventing bleeding. Liu et al. [155] recently reported a case of an ITP patient with the syndrome of spleen blood deficiency treated by a self-designed formula, “*Bupi Shengxue Decoction*” (*Spleen-Invigorating and Blood-Generating Decoction*), with the combined use of other herbs such as *Astragalus* and *White Atractylodes*. Four months of treatment led to a stabilization in the platelet count within the normal range for this patient. In clinical research, Yan et al. [156] reported that the *JYSD* formula could rapidly increase the peripheral blood platelet count of ITP mice, with earlier onset time than that of prednisone acetate. In addition, the *JYSD* formula can regulate the levels of cytokines such as IFN-γ, IL-2, IL-4, IL-10, TGF-β, IL-27, and IL-17A in ITP mice through the reversal of the Th1/Th2 and Th17/Treg immune imbalances possibly. Given that, Nan et al. [157] researched the formula for strengthening the spleen, boosting Qi and nourishing blood, which could regulate SDHA expression in ITP mice, thereby improving mitochondrial function that provide sufficient energy support for megakaryocyte differentiation and maturation. Evidence from animal experiments and omics analyses also documents that DBD can enhance peripheral hematopoiesis [158], suppress Th1/Th17 expansion, promote Treg differentiation, and down-regulate inflammatory pathways such as JAK/STAT. With respect to the above, these findings may provide a transferable mechanistic basis for the dual immuno-hematopoietic regulation underlying its potential therapeutic role in autoimmune bleeding disorders such as ITP.

#### Yiqi Tongyang Granules

*Yiqi Tongyang Granules* constitute a TCM for CITP and RITP treatment. Based on a modified *Fangguizhi Decoction* and *Sijunzi Decoction*, it is formulated and composed mainly of *Pseudostellaria heterophylla*, *stir-fried Atractylodes macrocephala*, *cinnamon twigs*, *Paeonia lactiflora*, *Cynomorium songaricum*, *Epimedium*, *honey-fried Glycyrrhiza uralensis*, etc. Initially, the team led by Yang et al. [159] discovered the critical role of *Yiqi Tongyang Decoction (YTD)* in CITP treatment by modulating the imbalance in T lymphocyte levels, particularly the Th1/Th2 and Th17/Treg cell imbalances. Recently, the same team reported significantly increased number of megakaryocytes in the bone marrow of the modeled mice; whereas treatment with *YTD* resulted in an obviously decreased total number of megakaryocytes. Critically, *YTD* intervention decreased the proportions of both primitive and immature megakaryocytes, but increased the proportion of plate-producing megakaryocytes. In addition, related literature review has also summarized the clinical evidence and potential mechanistic pathways underlying the application of *Yiqi Tongyang* formulas in ITP [6].

#### Shao Xian Yu Granules

*Shao Xian Yu Granules* are a compound composed of *white peony root*, *cypress Rhizoma* and *Cornus officinalis* based on the theory of TCM. It works to increase the platelet count and controls bleeding, which is highly important for improving the bleeding symptoms of ITP patients and protecting the spleen. Specifically, the *white peony root*, a TCM for nourishing blood, can consolidate liver Yin to soften the liver parenchyma, beyond nourishing the liver Yin to enrich liver blood. In line with the physiological characteristics of the liver mostly, it is a representative TCM for nourishing blood and softening the liver [160]. The occurrence of ITP may be attributed to the deficiency of the liver and spleen, manifesting as their failure of storing and regulating blood. Therefore, corresponding medication should consider the principle of tonifying the liver and spleen [161, 162]. Kou et al. [163, 164] reported that *Shao Xian Yu granules* could increase the platelet count and the number of platelet-producing megakaryocytes in ITP mice, and alleviate damage to the liver and spleen in a dose-dependent manner.

### Compounds primarily regulating T-cell immunity

These compounds primarily modulate T-cell subsets, cytokine profiles, and immunosuppressive functions, such as elevating Tregs, balancing Th1/Th2/Th17 ratios, as well as inhibiting excessive T- and B-cell activation. Eventually, it can contribute to a mitigation in autoimmune platelet destruction in ITP, thereby providing targeted intervention for immune-dysregulated syndromes.

#### Guipi decoction

*Guipi decoction* is a representative prescription for treating ITP syndrome caused by spleen Qi deficiency or Qi deficiency without control [165, 166]. In an early small-sample clinical study [167], a modified *Guipi decoction* was adopted to treat chronic ITP, with platelet elevation in seven of ten patients, accompanied by reduced platelet-associated IgG in most cases. Therefore, this formula may exert therapeutic effects by decreasing autoantibody burden and improving peripheral immune microenvironment. Jia et al. [168] reported improved bleeding symptom, TCM syndromes and fatigue when applying the modified *Guipi decoction* combined with Prednisone Acetate Tablets for ITP treatment, with the therapeutic effect increasing to 88.75% [136, 169]. Further supporting these findings, Li et al. [170] demonstrated that *Guipi decoction* could suppress T cell and B cell proliferation and activation by elevating Treg numbers and enhancing TGF-β1 expression, ultimately leading to increased platelet counts in a mouse model of ITP. In addition, Zheng et al. [104] reported that in the treatment of ITP, *Guipi decoction* combined with dexamethasone might promote M2-type macrophage polarization by inactivating the NF-κB signaling pathway, increasing IL-10 and 5-HT production, as well as inhibiting IL-12 and TNF-α expression.

#### Ningxue Shengban decoction

*Ningxue Shengban decoction* is a TCM prescription with multiple functional effects such as clearing heat and detoxifying, nourishing Yin and cooling blood, resolving blood stasis and stopping bleeding. Concerning its role of maintaining immune homeostasis, it can restore the Treg/Th17 balance and hinder inflammatory or differentiation-related signaling pathways such as PI3K-Akt-mTOR and Notch, and alleviate ITP-like immune dysregulation, thereby reducing proinflammatory cytokines (e.g., IL-6 and IL-17A) and improving the peripheral immune microenvironment. In animal and cellular models, *NXSB* could up-regulate Foxp3 while downregulating RORγt and STAT3, suppress inflammasome and NF-κB-related pathways, and attenuate inflammation, leading to increased platelet counts and restored Treg function in ITP mice[171]. Moreover, it can replenish the depleted Yin and blood. *Rhinoceros Horn and Rehmannia Decoction* is a common prescription for treating all bleeding symptoms caused by blood heat [172]. The addition of *Erzhi pills* can further prolong and stabilize the effect, in addition to enhancing the therapeutic effect of the *Rhinoceros Horn* and *Rehmannia Decoction*. Huang et al. [173] reported that *Ningxueshengban decoction* for nourishing yin, clearing heat and cooling blood also exhibited a significant therapeutic effect on adult ITP.

#### Rhinoceros Horn Rehmannia Decoction

*Rhinoceros Horn* and *Rehmannia Decoction* is composed of a *rhinoceros horn*, *raw rehmannia*, *red peony root* and *peony bark*. Such combination can restore the depleted Yin and blood, assist in clearing heat and cooling blood, relieve the heat caused by blood accumulation, nourish Yin and stop bleeding [174]. *Rhino Horn and Rehmannia Decoction* is a representative prescription for treating ITP with the syndrome involving blood heat, which is used for emergency treatment of severe cases given its effect of reducing the risk of bleeding [175]. He et al. [172] found that this formula could prevent the development of ITP by inhibiting TLR-4 expression through miR-181a. Wang et al. [176] reported that this formula had anti-inflammatory, antivascular endothelial proliferation, and antiplatelet activation effects. They can up-regulate Treg subsets, maintain the Treg/Th17 balance, and regulate IL-17 and IL-35 expression to exert immunomodulatory effects. Similarly, by employing network pharmacology, Li et al. [177] demonstrated that the active ingredients of *Rhinoceros Horn and Rehmannia Decoction*, such as quercetin and kaempferol, would treat ITP through multiple targets via the PI3K-Akt and IL-17 signaling pathways. In addition, Hu et al. [178] also supported the modified application of *Rhinoceros Horn* and *Rehmannia Decoction* for restoring the immune balance of Treg/Th17 cells by increasing the proportion of Treg cells, decreasing the proportion of Th17 cells, and regulating the secretion of IL-17, IL-35, and related cytokines, thereby inhibiting the inflammatory response and promoting platelet production.

#### Huangqi Jianzhong decoction

*Huangqi Jianzhong decoction* is a traditional prescription derived from the classic Chinese medical text “*Synopsis of the Golden Chamber*: Concurrent Treatment of Pulses and Symptoms of Blood Arthralgia and Debility”. Current Western medical research on *Huangqi Jianzhong decoction* primarily focuses on its role in eradicating *Helicobacter pylori* [179, 180], and the presence of *Helicobacter pylori* has been confirmed as a pathogenic factor in ITP [181]. Modern pharmacological research have documented an active role of *Huangqi Jianzhong decoction* in platelet formation and platelet destruction mitigation [182]. Rrecent reviews and experimental studies confirm that key active constituents derived from *Astragalus* (e.g., as astragaloside IV and methylated isoflavones) can regulate the Treg/Th17 balance, suppress NF-κB-mediated inflammatory signaling, and enhance mucosal barrier integrity, thereby providing pharmacological support for the application of *Huangqi Jianzhong decoction* in immune-mediated diseases [183]. Clinically, Ma et al. [184] adopted a modified therapeutic regimen involving *Huangqi Jianzhong decoction*, which could promote immune function recovery by regulating the Th17/Treg balance. Moreover, both experimental and clinical studies on ITP have identified that *Astragalus*-containing formulations could reduce bleeding frequency and optimize immune profiles, offering modern evidence for the “spleen-governing blood” concept that links immune regulation with hemostasis [146].

#### Spleen-strengthening, Qi-Tonifying and blood-regulating formulas

The formula for strengthening the spleen, tonifying Qi and regulating blood is developed based on the classic prescription of *Sijunzi Decoction*. The addition of *Astragalus membranaceus*, *donkey-hide gelatin* and *Rubia milii* can further enhance the effect of consolidating Qi and preventing bleeding. Liu et al. [155] recently reported a case of an ITP patient with the syndrome of spleen blood deficiency treated by a self-designed formula, “*Bupi Shengxue Decoction*” (*Spleen-Invigorating and Blood-Generating Decoction*), with the combined use of other herbs such as *Astragalus* and *White Atractylodes*. Four months of treatment led to a stabilization in the platelet count within the normal range for this patient. In clinical research, Yan et al. [156] reported that the *JYSD* formula could rapidly increase the peripheral blood platelet count of ITP mice, with earlier onset time than that of prednisone acetate. In addition, the *JYSD* formula can regulate the levels of cytokines such as IFN-γ, IL-2, IL-4, IL-10, TGF-β, IL-27, and IL-17A in ITP mice through the reversal of the Th1/Th2 and Th17/Treg immune imbalances possibly. Given that, Nan et al. [157] researched the formula for strengthening the spleen, boosting Qi and nourishing blood, which could regulate SDHA expression in ITP mice, thereby improving mitochondrial function that provide sufficient energy support for megakaryocyte differentiation and maturation. Evidence from animal experiments and omics analyses also documents that DBD can enhance peripheral hematopoiesis [158], suppress Th1/Th17 expansion, promote Treg differentiation, and down-regulate inflammatory pathways such as JAK/STAT. With respect to the above, these findings may provide a transferable mechanistic basis for the dual immuno-hematopoietic regulation underlying its potential therapeutic role in autoimmune bleeding disorders such as ITP.

#### Shengxue Xiaoban capsule (SXXBC)

*SXXBC* is a TCM compound guided by the principles of “clearing heat, cooling blood, resolving stasis, and regulating immunity”. It has been increasingly applied as an adjunctive therapy for ITP, with emerging studies exploring its underlying mechanisms. For instance, a meta-analysis encompassing 27 randomized controlled trials (RCTs) demonstrated that compared with glucocorticoid monotherapy, *SXXBC* combined with glucocorticoids could significantly improve the overall response rate and increase platelet counts [185]. *Indigo naturalis*, a major ingredient of this formula, can clear heat, detoxify, cool the blood and remove spots; moreover, its medicinal ingredient, *peony bark*, functions to cool the blood and prevent blood stasis. Liu et al. [186] treated ITP mice with *SXXBC*, with the observation of increased PLT count and elevated number of plate-producing megakaryocytes after intervention. Moreover, the intervened ITP mice were also detected with escalated composition ratio of Treg cells, Treg/CD4 ratio, and expression level of Foxp3 mRNA in the spleen. As proven by network pharmacology combined with experimental validation, by modulating signaling pathways such as PI3K-AKT and MAPK, the active components of *SXXBC* could promote megakaryocyte proliferation and differentiation, and ameliorate bleeding and immune phenotypes in ITP animal models [70].

### Compounds with hemostatic and blood-cooling properties

This category of compounds emphasizes hemostasis through mechanisms such as cooling blood, clearing heat, resolving stasis, and enhancing coagulation. Unlike other therapeutic classes, these compounds prioritize the control of bleeding symptoms, and support systemic blood harmony, which are highly suitable for ITP with blood-heat manifestations.

#### Liangxue Zhuyu Decoction

In the *Liangxue Zhuyu Decoction*, *water buffaloe horns*, *peach kernels,* and *Ligusticum chuanxiong* can promote blood circulation and ameliorate blood stasis. *Angelica sinensis* can boost blood circulation and blood production. *Raw Rehmannia glutinosa* and *Paeonia rupaeensis* can clear heat and cool blood. *Astragalus membranaceus* can clear heat, detoxify, tonify Qi and enrich blood. *Agaricus cassia* and *Gardenia jasminoides* have astringent and hemostatic effects. Raw *Sanguisorba officinalis* and raw *Platycladus orientalis* can cool blood and prevent bleeding. *Cornus officinalis* tonifies the liver and kidneys, while *peony* can nourish blood, consolidate Yin and calm the liver Yang. A combined use of these herbs works synergistically to achieve the effects of tonifying Qi and consolidating the constitution, clearing heat, promoting blood circulation, dispersing blood stasis and cooling the blood [187]. In evidence aligned with the “blood-cooling” therapeutic spectrum, Yin-nourishing and blood-cooling formulations combined with prednisone can increase platelet counts and markedly correct Th17/Treg imbalance in mouse ITP model. Mechanistically, the combined therapy could regulate the ST2/IL-33 signaling pathway, suggesting the experimentally verifiable biological plausibility of this “blood-cooling and immune homeostasis-restoring” mechanism [188]. Moreover, modern pharmacological research has confirmed that *Astragalus membranaceus* and *Paeonia suffruticosa* can enhance the immune function, *Angelica sinensis* can promote the recovery of spinal cord hematopoietic function, while *water buffaloe horns* can shorten the coagulation time and promote platelet aggregation [189]. Huang et al. [190] reported that *Liangxue Zhuyu decoction* could increase the PLT count, improve the immune function of patients and stop bleeding. Moreover, it was associated with a significantly lower incidence of adverse reactions in the observation group than that in the control group, indicating a superior safety of this to that of Prednisone Acetate Tablets.

Table 4 systematically summarize the mechanisms, molecular targets, types of evidence, and limitations of key hemostatic and blood-cooling TCM compounds ITP. It critically evaluate corresponding evidence by synthesizing existing studies, highlighting potential research gaps such as the scarcity of RCTss and the need for standardization.

**Table 4:** Critical evaluation of evidence for key TCM compound formulas in ITP treatment

Herbs/Formulas	Proposed Mechanisms	Key Molecular Targets/Cell Types	Type of Evidence	Remaining Questions/Limitations
*Jianpi Yiqi Shexue Decoction (JYSD)*	Improve hematological and coagulation parameters; and modulate the gut-brain axis for serotonin regulation to enhance coagulation	5-HT levels; megakaryocytes, and gut microbiota	Animal model, and clinical trial	Limited long-term follow-up in clinical settings; gut-brain axis links requiring validation in larger RCTs; and potential variability in herbal sourcing
*Yiqi Ziyin*	Modulate CD4⁺ T-cell differentiation to restore immune balance	PI3K-Akt pathway; and CD4⁺ T cells	Animal model, and in vitro	Predominantly preclinical evidence; the lack of head-to-head comparisons with standard immunosuppressants; and syndrome-specific applicability untested in heterogeneous ITP populations
*Jianpi Zishen Xiehuo Formula*	Regulate M1 macrophage polarization; and suppress JAK/STAT signaling to reduce Th1 dominance and platelet destruction	JAK/STAT pathway, NF-κB; M1 macrophages, and Th1/Th2 cells	Animal model, in vitro, and clinical trial	Inconsistent dosing across studies; limited data on RITP; and the absence of exploring potential herb-drug interactions with steroids
*Ejiao Siwu Decoction*	Exert multi-target action on immune modulation and hematopoiesis via identified active ingredients	PI3K-Akt, IL-17 pathways; megakaryocytes, and T cells	In vitro, and animal model	The lack of clinical translation, despite robust analytical methods (HPLC–MS); unclear efficacy in chronic vs. acute ITP; and requirement for the standardization of donkey-hide gelatin component
*Guipi Decoction*	Exert immunomodulatory effect via Treg elevation and NF-κB inhibition; possess anti-inflammatory effect to reduce bleeding and vascular damage	NF-κB pathway, TGF-β1, IL-6, IL-10; Tregs, and macrophages (M2 polarization)	Animal model, and clinical trial	High efficacy in Qi-deficiency syndrome but variable response rate; adverse reaction profiles in combination therapies requiring monitoring; and more RCTs needed for glucocorticoid-sparing effects
*Liangxue Zhuyu Decoction*	Clear heat, promote blood circulation, and enhance immune function to elevate platelet counts and halt bleeding	Coagulation factors; immune cells, and platelets	Clinical trial, and animal model	Unclear mechanisms beyond coagulation, despite superior safety over prednisone; and the lack of dosage optimization for severe bleeding cases
*Ningxue Shengban Decoction*	Nourish Yin, clear heat, resolve stasis; and adjust Th1/Th2 imbalance for autoimmune regulation	Th1/Th2 cytokines; T cells, and platelets	Clinical trial (RCT)	No recurrence rates post-treatment reported, despite its efficacy in reducing hormone dosage; and requirement for mechanistic dissection concerning component synergies (e.g., *Erzhi Pills* addition)
*Modified Siwu Decoction*	Nourish blood without stasis; alleviate megakaryocyte pathology, and reduce platelet-associated antibodies	Platelet antibodies; megakaryocytes, and blood cells	Animal model, and clinical trial	Small human trial scale, despite its comparability to prednisone in models; and requirement for further clarification of the role of wind-dispelling additions in depression syndromes
*Rhinoceros Horn Rehmannia Decoction*	Inhibit TLR-4 via miR-181a; and exhibit anti-inflammatory and antiplatelet activities to maintain Treg/Th17 balance	TLR-4, miR-181a, IL-17, IL-35; Tregs/Th17 cells, and endothelial cells	Animal model, and network pharmacology	Ethical concerns with rhinoceros horn substitutes (water buffalo); limited indication of blood-heat syndrome, even with promising anti-vascular effect; and the requirement for more clinical data on bleeding risk reduction

## Research on the integrated treatment of ITP with TCM and Western medicine

**Fig. 5:**
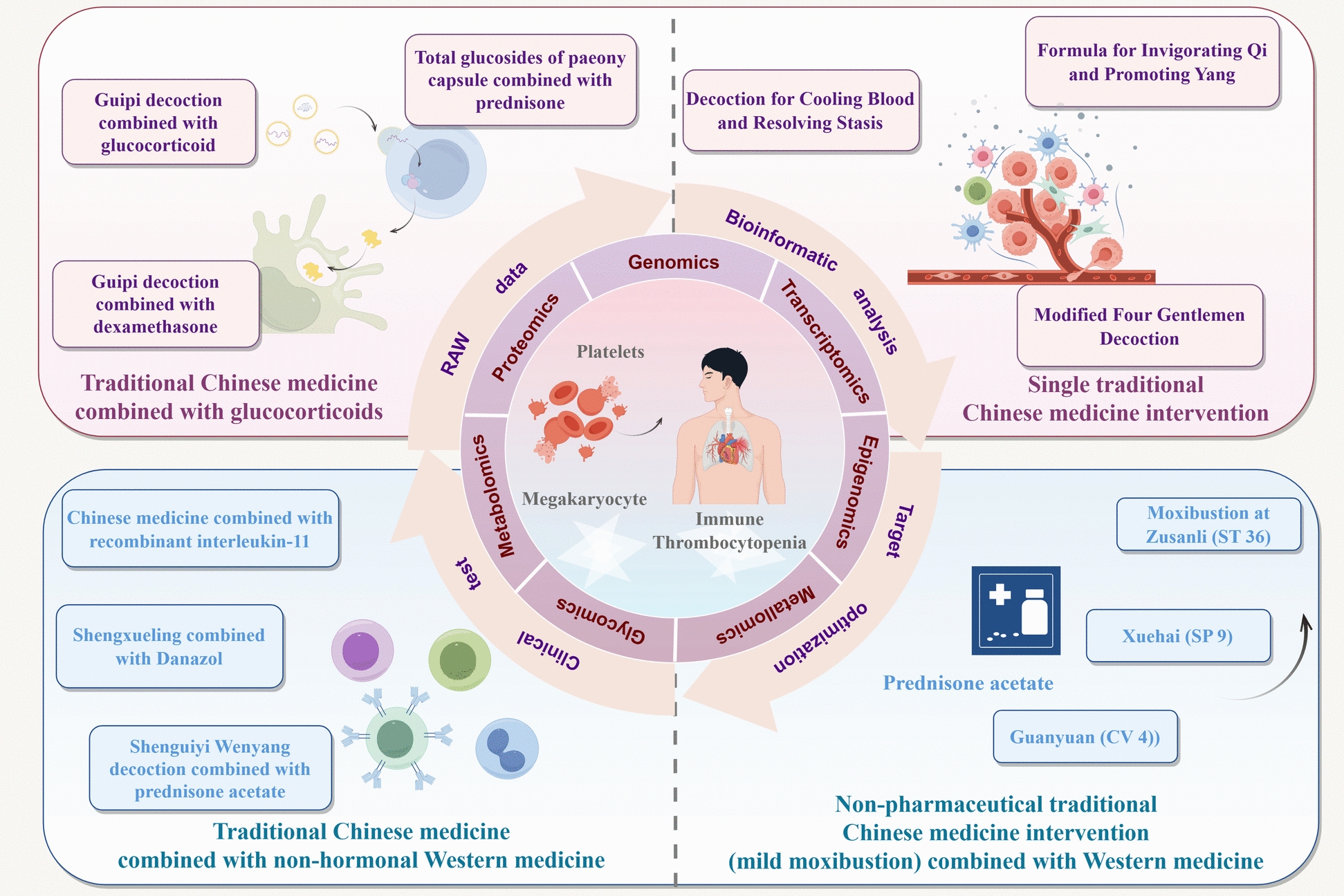
Research on the integrated use of TCM and Western Medicine for ITP treatment. This diagram summarizes integrated therapeutic strategies incorporating TCM and Western medicine. It includes TCM preparations in combination with glucocorticoids, dexamethasone, recombinant interleukin-11, danazol, or prednisone acetate. Non-pharmaceutical TCM modalities such as moxibustion are also illustrated, along with herbal formulas reinforcing Qi and Yang, demonstrating a multi-level approach to managing ITP

In the field of modern medicine, researchers have witnessed the continuous emergence of new drugs for ITP treatment, with promising therapeutic effect. However, there are still problems such as long disease course, high cost, significant adverse reactions, and risk of drug resistance. TCM, with similar functions of increasing platelet counts and maintaining their stability, has good therapeutic effects in alleviating patients' clinical symptoms, improving their quality of life, reducing the adverse reactions of Western medicine. Therefore, the integrated treatment of ITP with TCM and Western medicine has gradually shown its advantages in clinical settings. Figure 5 summarizes studies on this integrated approach, depicting synergistic pathways and clinical outcomes that combine immunomodulatory effects of TCM with Western medicine to enhance efficacy and reduce side effects. Meanwhile, Table 5 compiles relevant studies on integrated treatment, highlighting therapeutic options, indications, efficacy measures, mechanisms, and key findings to provide an evidence-based overview of the proposed integrated therapies.

**Table 5:** Relevant studies on the application of integrated TCM and Western medicine for the treatment of ITP

Treatment options	Types of indication	Core efficacy measures	Mechanisms of action	Key findings
TCM: *Purpura Decoction*; WM: rhTPO	Yin deficiency and fire hyperactivity (glucocorticoid-unresponsive)	Total effective rate: 64.52%; Platelet count: Significantly increased; Bleeding symptom: Significant improvement	Not clarified	Purpura decoction combined with rhTPO could effectively improve the bleeding symptoms and increase the platelet count in ITP patients with the syndrome of Yin deficiency and fire hyperactivity who were unresponsive to glucocorticoid
TCM: *Total Glucosides of Paeony Capsule*; WM: Prednisone	–	Total effective rate: 45% (treatment group); Platelet count: Significantly increased (treatment group); Slightly increased bone marrow megakaryocyte (treatment group); T cell subsets: Significantly decreased CD4^+^ T cells, and slightly increased CD8 + T cells in the treatment group	Adjust the disorder of T cell subsets	Total glucosides of paeony capsules combined with prednisone could increase the number of platelets in patients with ITP, improve the abnormality of megakaryocytes in bone marrow, and correct the imbalance of T cell subsets
TCM: *Shengui-Yiqi Wenyang Decoction*; WM: Conventional treatment	–	The total effective rate: 95.12% in the observation group Platelet count: Increased significantly in the observation group Safety: Higher in the observation group	Not clarified	Shengui-yiqi Wenyang decoction combined with western medicine (prednisone acetate, etc.) was effective and safe in the treatment of RITP
TCM: *Xiaochaihu Decoction*; WM: Glucocorticoid + Vitamin C	–	Total effective rate: Significantly higher in the observation group than the control group Short-term effective rate: Significantly higher in the observation group than the control group Adverse reactions: Significantly lower in the observation group than the control group	Not clarified	Xiaochaihu decoction combined with glucocorticoid and vitamin C could improve the efficacy of ITP and reduce the adverse reactions of conventional treatment
TCM: Self-made Purpura No.1 Prescription; WM: Methylprednisolone Sodium Succinate Injection	Blood-heat and wind excess	Total effective rate: 86.66% in the treatment group (not clear in the control group); Platelet count: increased more effectively in the treatment group; Bleeding symptoms: Significantly reduced in the treatment group; -Significantly shortened duration of glucocorticoid use in the treatment group	Not clarified	Self-made Purpura No. 1 decoction combined with methylprednisolone sodium succinate was more effective in the treatment of ITP with the syndrome of blood-heat and wind excess, and could shorten the duration of hormone use
TCM: *Shengplatelet Capsule* (SXT); WM: Prednisone	–	Platelet count: Significantly higher in the observation group than the control group; Treg cell count: Significantly higher in the observation group than the control group; IL-10 contents: Significantly higher in the observation group than the control group Total effective rate: Significantly better in the observation group than the control group	Regulate the immunity by changing the content of Treg cells	SXT combined with prednisone could improve the platelet level and efficacy of ITP patients by increasing Treg cell count and IL-10 content
TCM: *Yiqi Ziyin Prescription*; WM: Conventional treatment	–	Total effective rate: Significantly better in the treatment group than the control group The number of Treg: Increased significantly in the treatment group -Th17 expression: Significantly reduced in the treatment group	Restore the function of Treg, and down-regulate the expression of Th17	Yiqi Ziyin decoction combined with conventional Western medicine was more effective in the treatment of newly diagnosed ITP, which could regulate Treg/Th17 balance
TCM: Cuscutazi Drink; WM: Glucocorticoid	–	Platelet-related antibody: Decreased in both groups, and more prominent in the combination group Th1/Th2 balance: improved in both groups, and better regulatory effect in the combined group	Balance Th1/Th2 and reduce platelet-associated antibodies	The effect of Cuscutazi decoction combined with glucocorticoid was better in regulating Th1/Th2 balance and platelet-related antibodies than those of glucocorticoid alone in patients with ITP
TCM: *Shengxueling*; WM: Low-dose glucocorticoid (Control: Conventional-dose glucocorticoid)	–	Total effective rate: 83.33% in the observation group vs 60.00% in the control group; Platelet count: Increased significantly in the observation group Fatigue state: Significantly relieved in the observation group 5-HT level: Decreased more significantly in the observation group	Decrease 5-HT concentration, alleviate fatigue and increase platelet count	Shengxueling combined with low-dose glucocorticoid was better than conventional dose glucocorticoid alone in the treatment of chronic ITP, and could relieve fatigue by reducing 5-HT
TCM: *Qihuang Decoction*; WM: Glucocorticoid	–	Total effective rate: 86.67% in the treatment group vs 68.89% in the control group; Recurrence rate: 10.26% in the treatment group vs 29.03% in the control group; Immune balance: Significantly improved in the treatment group	Regulate cytokines and improve the expression of PD-1/PD-L1 signaling in CD4 + T lymphocytes	Qihuang decoction combined with glucocorticoid exhibited better efficacy and lower recurrence rate in the treatment of ITP, and could improve the immune balance by regulating PD-1/PD-L1 signaling
TCM: *Shengxueling*; WM: Danazol		Clinical symptoms: Significantly improved in the experimental group; Higher total effective rate of the experimental group than that of the control group Adverse reactions: No obvious adverse reactions in the experimental group Telomere attrition in peripheral blood leukocytes: Partial improvement in both groups	No specific mechanism identified (referring to improved leukocyte telomere attrition)	Shengxueling combined with danazol was effective and safe in the treatment of ITP, which could improve telomere attrition in white blood cells
TCM: *Modified Yin-nourishing and Blood-cooling Formula*; WM: Glucocorticoid	Yin deficiency and fire hyperactivity (severe)	Platelet level: Significantly increased in the experimental group Bleeding symptoms: Significantly improved in the experimental group; Significantly lower recurrence rate of the experimental group than that of the control group Immune disorders: Significantly improved (Th17/Treg imbalance, inflammatory cytokines, humoral immunity, and platelet-associated antibodies) in the experimental group	Regulate the imbalance of Th17/Treg, reduce inflammatory cytokines and platelet-related antibodies, and improve humoral immunity	Modified Yiyin Liangxue decoction combined with glucocorticoid could increase platelet count, improve bleeding, reduce the recurrence rate, and treat immune disorders in the treatment of severe ITP with the syndrome of Yin deficiency and fire hyperactivity
TCM: *Yinqiao Shengban Prescription*; WM: Hormone	Wind-heat injuring collaterals	Bleeding symptom score: Lower in the treatment group than the control group Platelet count: Higher in the treatment group; TCM syndrome score: Improved more significantly in the treatment group Total effective rate: Better in the treatment group than that in the control group	Improve the hypocoagulable state	Yinqiaoshengbian decoction combined with hormone could significantly improve bleeding symptoms, platelet, and TCM syndrome efficacy in the treatment of ITP with the syndrome of wind-heat injuring collaterals
TCM: Acupuncture (ST36, SP10, CV4) + Prof. Yao Naizhong's Empirical Formula; WM: Same unspecified WM	Kidney essence deficiency (chronic)	Platelet level: Significantly increased in the treatment group; Reticulocyte ratio: Decreased significantly in the treatment group Bleeding symptoms: Significantly reduced in the treatment group; Total effective rate: Increased significantly in the treatment group	Not clarified	Acupuncture and moxibustion combined with Professor Yao Naizhong's empirical formula and western medicine were better than simple western medicine in the treatment of chronic ITP with the syndrome of kidney essence deficiency
TCM: *Erzhi Shengban Decoction*; WM: Cyclosporine (Control: Cyclosporine)	CITP	Total effective rate: 74.29% in the treatment group, significantly lower than that in the control group (not clear) Platelet count: Significantly increased in the treatment group; Recurrence rate: Significantly lower in the treatment group	Not clarified	Erzhishengban decoction was more effective than cyclosporine in the treatment of chronic ITP, accompanied by lower recurrence rate
TCM: Qi-invigorating, Yin-nourishing and Blood-nourishing Formula; WM: Conventional treatment	CITP	PLT count: Significantly increased; Bleeding: Significantly improved; Steroid withdrawal rate: 86.4%; HRQoL (quality of life): Significantly improved in some domains; Fatigue symptoms: Significantly improved; Adverse reactions: Fewer than the control group	Not clarified	Yiqi Yangyin and Xuefang could increase platelet count, improve bleeding and fatigue, help to reduce hormones, and improve the quality of life in the treatment of chronic ITP
TCM: *Xiaochao Xiaochuan Decoction*; WM: Conventional treatment	Pediatric ITP	Total effective rate: 84.4% in the observation group vs 62.5% in the control group; PAIgA, PAIgG, PAIgM levels: Significantly lower in the observation group	Reduce platelet-associated antibodies (PAIgA, PAIgG, and PAIgM)	Xiaochao Xiaochuan decoction was superior to conventional treatment in the treatment of children with ITP, and could significantly reduce platelet-related antibodies
TCM: Modified Liuwei Dihuang Pill (with *Astragalus, Rehmannia*, Angelica); WM: Conventional treatment	–	Total effective rate: Good efficacy; Immune function: Immunomodulatory effect of *Astragalus membranaceus*; Bone marrow hematopoiesis: Accelerated proliferation and differentiation of hematopoietic cells by *Rehmannia radix* and *Angelica radix*	Regulate immunity and promote the proliferation and differentiation of bone marrow hematopoietic cells	Jiawei Liuwei Dihuang Pill was effective in the treatment of ITP, and its mechanism was related to immune regulation and the promotion of bone marrow hematopoiesis
TCM: *Spleen-invigorating, Kidney-nourishing and Fire-reducing Formula*; WM: Conventional treatment	–	Platelet count: Significantly increased; Immune balance: Restored Th17/Treg balance	Regulate Th17/Treg immune balance	Jianpi-zishenxiehuo decoction could improve platelet count and restore Th17/Treg balance in the treatment of ITP
TCM: *Yiqi Shengxue Formula*; WM: Conventional treatment	–	CD4 + , CD4 + CD25 + , IL-4 T lymphocytes: Increased expression; CD8 + , IFN-γ, IL-17 T lymphocytes: Decreased expression	Regulate T lymphocyte subsets and cytokines (IL-4, IFN-γ, and IL-17)	Yiqi Shengxue Decoction could improve the immune disorder of ITP by regulating the levels of peripheral blood T lymphocyte subsets and cytokines
TCM: Mild Moxibustion (ST36, SP9, CV4); WM: Prednisone Acetate	–	Platelet count: Significantly increased in the combination group; Bleeding symptoms: Significantly improved in the combination group; Physical strength and quality of life: Significantly improved in the combination group	Regulate serum IL-10 and IL-33 levels	Mild moxibustion combined with prednisone acetate was superior to simple hormone therapy in the treatment of ITP, which could improve symptoms by regulating IL-10 and IL-33
TCM: *Guipi Decoction*; WM: Hormone	Qi dysfunction in blood control	Platelet count: Rapidly increased in the combination group; TCM syndromes: Better improvement in the combination group;	Not clarified	Guipi decoction combined with hormone could rapidly increase platelet count in the treatment of ITP with the syndrome of Qi dysfunction in blood control
TCM: *Yiqi Tongyang Decoction vs Guipi Decoction*; WM: Conventional treatment	CITP	TCM syndromes: More significantly improved by the Yiqi Tongyang prescription; Immune function: Better regulation by the Yiqi Tongyang formula Platelet count: Increased more significantly by the Yiqi Tongyang decoction Clinical symptoms: Alleviated more obviously by the Yiqi Tongyang decoction	Not clarified	Yiqi Tongyang decoction was better than Guipi decoction in the treatment of CITP
TCM: *Huangqi Jianzhong Decoction*; WM: Hormone	–	T lymphocyte subsets: Improved significantly in the combination group; Immune function: Improved significantly in the combination group	Improve T lymphocyte subsets and regulate immune function	Huangqi Jianzhong decoction combined with hormone could significantly improve T lymphocyte subsets and immune function in the treatment of ITP
TCM: *Spleen-invigorating, Qi-invigorating and Blood-stasis-resolving Granule*; WM: Hormone	–	Total effective rate: Better in the combined group than that in the simple hormone group; TCM syndromes (fatigue, and burnout): Significantly improved in the combination group; Platelet level: Significantly increased in the combination group; T lymphocyte subsets: Significantly regulated in the combination group	Regulate T lymphocyte subsets	Jianpi Yiqi Yuxue granule combined with hormone was superior to simple hormone in the treatment of ITP, which could improve TCM syndromes and T lymphocyte subsets
TCM: *Dijincao Tablets*; WM: Conventional treatment	–	T lymphocyte subsets: Corrected disorder in the combined group; The level of cytokines: Significantly lower in the combined group; Platelet level: Significantly increased in the combined group;	Regulate the proportion of T lymphocyte subsets and the levels of cytokines	Dijincao tablets combined with conventional treatment could play an important role in the treatment of ITP by regulating lymphocyte subsets and cytokines
TCM: *Blood-cooling and Blood-stasis-resolving Decoction*; WM: Prednisone Acetate Tablets	–	Platelet level: Significantly increased in the Liangxue-Zhuyu decoction group; Bleeding symptoms: Significantly improved in the Liangxue-Zhuyu decoction group; Hormone adverse reaction: No adverse reaction in the Liangxue-Zhuyu decoction group (with adverse reaction in the control group)	Not clarified	Liangxue-zhuyu decoction was effective in the treatment of ITP, and had no adverse reactions of hormones, which was worthy of clinical application
TCM: *Modified Cijiao Dihuang Decoction*; WM: Conventional hormone therapy	–	The levels of CD3^+^ and CD4^+^: Significantly higher in the combined group than those in the simple hormone group; CD8^+^ level: Significantly lower in the combination group than the single hormone group; Symptoms and signs: Significantly improved in the combination group;	Regulate T lymphocyte subsets (CD3^+^, CD4^+^, and CD8^+^)	Cijiao Dihuang decoction combined with hormone could improve symptoms and regulate T cell subsets in the treatment of ITP
TCM: *Guipi Decoction*; WM: Dexamethasone	–	Total effective rate: Higher in the combined group than that of the simple hormone group Clinical symptoms (fatigue, dizziness, and bleeding): Better improvement in the combination group	Not clarified	Guipi decoction combined with dexamethasone was superior to dexamethasone alone in the treatment of ITP, and it was better at improving clinical symptoms
TCM: TCM (*Rehmannia, Astragalus*, *Buffalo Horn*, etc., modified by syndrome differentiation); WM: Recombinant IL-11 (Control: Recombinant IL-11)	RITP	Total effective rate: Higher in the combination group The platelet count: Higher in the combined group than that of the combined group	Not clarified	The efficacy of not clarified Chinese medicine combined with recombinant interleukin-11 was significantly better than that of recombinant interleukin-11 alone in the treatment of refractory ITP
TCM: *Modified Sijunzi Decoction*; WM: Conventional treatment	ITP mouse model	Platelet count: Significantly increased; Mean platelet volume: Significantly decreased; Plasma TPO and GP-IIb/IIIa: Significantly increased; Th17/Treg balance: Significantly regulated	Regulate the balance of Th17/Treg cells and up-regulate the contents of TPO and GP-IIb/IIIa	Jiawei Sijunzi decoction could increase platelet count, TPO, and GP-IIb/IIIa levels by regulating Th17/Treg balance in ITP mice

### Treatment of ITP with TCM combined with glucocorticoids

It has been confirmed previously that TCM combined with glucocorticoids can exert a synergistic effect by regulating immunity, improving symptoms and increasing the platelet count [191]. Jing et al. [192] compared the application of modified *Guipi decoction* combined with hormones and hormones alone in the treatment of primary ITP patients with the syndrome of Qi deficiency and blood stasis. They reported that the integrated treatment could rapidly increase the platelet count of patients. Song et al. [193] evaluated the use of the modified Ziyin Cooling Blood formula combined with hormones in the treatment of patients suffering from severe primary ITP with the syndrome of Yin deficiency and fire hyperactivity. They found better outcomes such as clinical bleeding symptom, platelet count and onset time in the experimental group than those in the control group. Similarly, several other studies have reported enhanced efficacy when combining TCM with conventional hormone therapy. For instance, in a retrospective analysis on ITP patients by Ma et al. [184], a modified *Huangqi Jianzhong decoction* plus hormones significantly improved T lymphocyte subsets and immune function compared to hormones alone. Additionally, Wu et al. [194] also confirmed superior therapeutic effects of *JYSD granules* combined with hormones in improving fatigue, listlessness, and platelet levels via regulating T lymphocyte subsets.

Furthermore, Zeng et al. [195] demonstrated the indicating immunomodulatory effect of a modified *Xi Jiao Di Huang decoction* added to conventional hormone therapy for 4 weeks, yielding significantly increased CD3^+^ and CD4^+^ T cells, and decreased CD8^+^ T cells. Wang et al. [196] reported that *Guipi decoction* combined with dexamethasone for 4 weeks could achieve higher effective rate and better improvement in fatigue, dizziness, and bleeding symptoms. Cao et al. [197] noticed that an integrated use of *white peony total glycoside capsules* and prednisone could also remarkably increase platelet counts, reduce bone marrow megakaryocytes, and modulate CD4^+^ and CD8^+^ T-cell subsets.

### Treatment of ITP with TCM and nonhormonal Western medicine

TCM combined with nonhormonal Western medicines is highly effective in treating RITP and hormone-ineffective ITP. Wang et al. [198] used recombinant IL-11 for the control group and administered TCM herbs (*Rehmannia glutinosa*, *Astragalus membranaceus*, *water buffaloe horns*, *Codonopsis pilosula*, *Atractylodes macrocephala*, *Angelica sinensis*, *Scrophularia ningpoensis*, purple pearl grass, *Paeonia lactiflora*, *Prunus mume* charcoal, and honey-fried liquorice) in the observation group. *Curculus orchioides*, deer horn gum, and *Epimedium* were added for individuals diagnosed with Yang deficiency. After two months of treatment, the observation group was observed with higher total effective rate and more platelet count than those in the control group. Xia et al. [199] conducted a RCT on 62 ITP patients with the syndrome of Yin deficiency and fire hyperactivity who were unresponsive to glucocorticoids. Consequently, *Ziyuan decoction* combined with recombinant human thrombopoietin yielded a total effective rate of 64.52%, which could significantly improve the bleeding symptom of patients and increase their platelet counts. Similarly, in a study on newly diagnosed ITP patients, Qian et al. [200] adopted *Yiqi Ziyin Formula* for treatment, with significantly better therapeutic effect in the treatment group than that in the control group, with increased number of Tregs, restored function and reduced number of Th17 cells.

Moreover, Wu et al. [201] divided 54 ITP patients into an experimental group (n = 36, *Shengxuelin* combined with danazole) and a control group (n = 18, danazole alone). The combined therapy realized improved clinical symptoms, greater therapeutic effects and no obvious adverse reactions. Additionally, concerning non-pharmaceutical TCM, Xiang et al. [202] included 100 patients of chronic ITP with the syndrome of kidney essence depletion, and noticed that acupuncture combined with Western medication could significantly increase platelet count, alleviate bleeding and improve the therapeutic effect.

### TCM-based ITP mechanisms and challenges

Specific TCM interventions are tailored to address distinct pathological imbalances in ITP. Based on the theory of TCM, these medicinal interventions are hypothesized to restore internal homeostasis through targeted effects on immunomodulation and hematopoiesis.

For instance, heat-clearing herbs, such as those in *Liangxue Zhuyu Decoction*, can cool the blood, detoxify pathogens, and modulate inflammatory cytokines to stabilize vascular integrity and reduce immune-mediated platelet clearance, which are theorized to counteract blood-heat patterns that drive erratic bleeding and platelet destruction [203, 204]. In contrast, Yin-nourishing herbs, exemplified in formulas like *Erzhishengban Decoction*, are postulated to replenish Yin deficiency, a disorder that may trigger internal heat and impaired megakaryocyte function, by enriching body fluids, harmonizing the liver and kidney meridians, and enhancing immunosuppressive T-cell subsets to curb excessive immunity and promote platelet survival [41, 205]. Qi-tonifying interventions, such as *Yiqi Tongyang Formula*, are hypothesized to correct Qi deficiency underlying spleen failure to control bleeding by strengthening spleen Qi, improving hematopoietic stem cell proliferation, and rebalancing Th1/Th2 cytokine profiles, thereby preventing bleeding and supporting long-term immune tolerance [159, 206]. Collectively, all these mechanisms can be empirically tested using biomarkers such as cytokine levels and platelet counts in controlled trials.

Noticeably, several challenges must be addressed to ensure the safety and efficacy of TCM with Western medicine in ITP management, despite its clinical benefits. Herb-drug interactions remain a concern, particularly when TCM formulations are used in combination with immunosuppressants (e.g., cyclosporine or glucocorticoids). Components such as *Astragalus* or licorice may potentially alter drug metabolism and increase toxicity by modulating cytochrome P450 enzymes [207, 208]. Meanwhile, it is a challenge to employ dosage individualization practically, given that TCM prescriptions based on syndrome differentiation often require dynamic adjustment, which may lead to variability in therapeutic exposure and response, especially in heterogeneous ITP populations. It is also povital to implement clinician education as many Western medicine-trained practitioners are unfamiliar with TCM principles, which may result in suboptimal prescription or oversight of contraindications.

In addition, AI enables the construction of graph neural networks for herb-target prediction and formula optimization, which is increasingly enhancing network pharmacology, and contribute to the addressing of the complexity of TCM through integrated multi-source data analyses [209]. However, the application of such approaches still has several limitations, such as database inconsistencies, algorithmic biases, and a reliance on computationally predicted interactions that lack experimental validation. Therefore, these computational approaches should be regarded as hypothesis-generating tools, rather than methods yielding definitive mechanistic proof [210]. It highlights the necessity for adopting interdisciplinary training programs to foster evidence-based integrative care. Eventually, with targeted addressing of these issues through standardized protocols, pharmacovigilance systems, and collaborative education, the integration of TCM and Western medicine will be optimized to support a holistic and scientifically grounded approach for the treatment of ITP.

## Future prospects

Currently, encouraging advancements have been achieved in the treatment of ITP by applying TCM. However, several persistent challenges may still impede its evidence-based integration into mainstream clinical practice. A strategic, evidence-informed roadmap is outlined here in response to these limitations and to promote translational progress, with an emphasis on hypothesis-driven research. Current mechanistic studies of TCM in ITP have predominantly reported associative rather than causal relationships. It implies the significance of targeted functional assays to advance this field. For example, CRISPR-Cas9-mediated gene editing can be employed to disrupt key signaling nodes such as JAK2 in CD4⁺ T cells, thereby validating the mechanistic role of compounds (e.g., Icaritin) in suppressing inflammatory signaling and enhancing thrombopoietin production [68, 211]. Simultaneously, Cdc42/RhoA signaling may regulate cytoplasmic maturation and proplatelet formation independent of polyploidization [212–214]. Therefore, complementary gain-of-function studies, such as overexpressing Cdc42 in human megakaryocyte cultures, may contribute to the clarification of its role in facilitating endomitosis through microtubule reorganization. Furthermore, there is so far insufficient evidence owing to existing small-scale studies and heterogeneous outcome measures, which undermine the generalizability of findings. There is a clear need for multicenter, prospective RCTs that evaluate, for instance, the application of Guipi Decoction combined with thrombopoietin receptor agonists in refractory ITP. Such trials should adopt standardized endpoints, such as sustained platelet stability, bleeding grade improvement, and corticosteroid dose reduction, through blinded central review and intention-to-treat analysis [6]. Meanwhile, variability in TCM preparation profiles may also compromise the reproducibility and clinical reliability. It proposes a requirement for high-resolution chromatography-mass spectrometry protocols to enable precise quantification of bioactive markers. Bioassay-guided fractionation can benefit the definition of critical quality attributes, such as icariin content thresholds in *Epimedium* extracts, to ensure consistent potency and batch-to-batch equivalence. Moreover, there is currently a lack of well-defined protocols for assessing synergistic efficacy and safety of the integrated therapeutic regimens of TCM and Western medicine. Future research should prioritize adaptive pharmacokinetic modeling to evaluate herb-drug interaction dynamics [215, 216]. In addition, predictive computational tools, such as network pharmacology, hold considerable potential for the optimization of TCM formulas, which, however, are frequently constrained by fragmented data resources. Corresponding predictive ability for multi-target effects can be improved by leveraging machine learning algorithms trained on integrated herb-target interaction repositories. These predictions should be systematically validated against experimental data to mitigate biases, such as epigenetic oversights [217–219].

In summary, it aims to shift the research paradigm of ITP treatment using TCM toward actionable and interdisciplinary frameworks that foster regulatory acceptance and improve clinical outcomes through rigorous and innovative approaches.

## Data Availability

No datasets were generated or analysed during the current study.

## Abbreviations

5-HT: 5-Hydroxytryptamine
ACE2: Angiotensin converting enzyme 2
ADP: Adenosine diphosphate
AI: Artificial intelligence
AMPK: Adenosine 5-monophosphate-activated protein kinase
APS: Astragalus polysaccharide
ASP: Angelica sinensis polysaccharides
ATP: Adenosine triphosphate
BAFF: B-cell activating factor
BCR: B-cell receptor
CCL2: Chemokine ligand 2
CLEC-2: C-type lectin-like receptor 2
CPP: Codonopsis pilosula polysaccharide
CXCL4: C-X-C motif chemokine ligand 4
Foxp3: Forkhead box P3
GADA-1: Anti-glutamic acid decarboxylase antibody
GATA-3: GATA binding protein 3
GC: Germinal center
GSK3β: Glycogen synthase kinase-3β
HIF-1α: Hypoxia-inducible factor-1 α
Hippo: Hippopotamus
IDO: Indoleamine 2, 3-dioxygenase
IFN-γ: Interferon-γ
Ig: Immunoglobulin
IL-21: Interleukin-21
IRS1: Insulin receptor substrate 1
ITP: Immune thrombocytopenia
JAK: Janus kinase
JYSD: Jianpi Yiqi Shexue decoction
LKB1: Live kinase B1
LLPCs: Long-life plasma cells
MS: Multiple sclerosis
mTOR: Mammalian target of rapamycin
MyD88: Myeloid differentiation primary response 88
NF-E2: Nuclear factor, erythroid 2
NF-κB: Nuclear factor kappa-B
PARs: Protease-activated receptors
PBMCs: Peripheral blood mononuclear cells
PD-L1: Programmed cell death ligand 1
PF4: Platelet factor 4
PGI2: Prostaglandin I2
PI3K: The phosphatidylinositol 3-Kinase
RANTES: Regulated upon activation normal T cell expressed and secreted
SDHA: Succinate dehydrogenase complex subunit A
STAT3: Signal transducer and activator of transcription 3
SXXBC: Shengxue Xiaoban capsule
TCM: Traditional Chinese medicine
Tfh: Follicular helper T
TGF-β: Transforming growth factor-β
TLRs: Toll-like receptors
TNF-α: Tumor necrosis factor-α
TPO: Thrombopoietin
UCHL5: Ubiquitin C-terminal hydrolase L5
UDCA: Ursodeoxycholic acid
VWF: Von Willebrand factor
YTD: Yiqi Tongyang decoction
